# Genome-wide characterization reveals complex interplay between TP53 and TP63 in response to genotoxic stress

**DOI:** 10.1093/nar/gku299

**Published:** 2014-05-13

**Authors:** Simon S. McDade, Daksha Patel, Michael Moran, James Campbell, Kerry Fenwick, Iwanka Kozarewa, Nicholas J. Orr, Christopher J. Lord, Alan A. Ashworth, Dennis J. McCance

**Affiliations:** 1 Centre for Cancer Research and Cell Biology, Queen's University Belfast, Belfast BT9 7BL, UK; 2 The Breakthrough Breast Cancer Research Centre, Institute for Cancer Research, Chelsea, London SW3 6JB, UK

## Abstract

In response to genotoxic stress the TP53 tumour suppressor activates target gene expression to induce cell cycle arrest or apoptosis depending on the extent of DNA damage. These canonical activities can be repressed by TP63 in normal stratifying epithelia to maintain proliferative capacity or drive proliferation of squamous cell carcinomas, where TP63 is frequently overexpressed/amplified. Here we use ChIP-sequencing, integrated with microarray analysis, to define the genome-wide interplay between TP53 and TP63 in response to genotoxic stress in normal cells. We reveal that TP53 and TP63 bind to overlapping, but distinct cistromes of sites through utilization of distinctive consensus motifs and that TP53 is constitutively bound to a number of sites. We demonstrate that cisplatin and adriamycin elicit distinct effects on TP53 and TP63 binding events, through which TP53 can induce or repress transcription of an extensive network of genes by direct binding and/or modulation of TP63 activity. Collectively, this results in a global TP53-dependent repression of cell cycle progression, mitosis and DNA damage repair concomitant with activation of anti-proliferative and pro-apoptotic canonical target genes. Further analyses reveal that in the absence of genotoxic stress TP63 plays an important role in maintaining expression of DNA repair genes, loss of which results in defective repair.

## INTRODUCTION

The TP53 family of transcription factors comprises three ancestrally conserved members *TP53*, *TP63* and *TP73*, which play critical roles in development, growth control, differentiation, cellular homeostasis and response to genotoxic and other types of stress (reviewed in (1,2)). While many overlapping functions have been described for TP53 family members, knockout studies and germline mutations reveal strikingly distinct phenotypes (3,4). *Trp53* knockout mice and patients with germline *TP53* mutations (Li-Fraumeni syndrome—LFS) (4,5) are highly cancer prone, whereas *Trp63* deficiency is perinatally lethal (3,6) due to a lack of skin and other developmental defects that are shared by patients with a spectrum of syndromes associated with *TP63* mutations (7).

TP53 family members share highly conserved core DNA binding and oligomerization domains, which facilitate tetramerization and DNA binding to TP53 response elements consisting of two decamers ‘RRRCWWGYYY’ separated by spacers of variable length (8). Target specificity is still poorly understood, but can be achieved through differential binding of family members and their respective isoforms to variable response elements to modulate transcription through intra- and inter-molecular interactions and post-translational modifications.

The transcriptional program regulated by TP53 represents a potent tumour suppressive pathway, which regulates expression of a network of genes in response to a range of stresses to induce cell cycle arrest, DNA repair, senescence or apoptosis (9). As such, TP53 is seen as a critical barrier to tumourigenesis and loss of wild-type p53 function occurs in more than half of all tumours through either mutation or compromised function caused by a variety of mechanisms including viral oncoproteins such as Human Papilloma Virus E6 (10) or overexpression of negative regulators such as MDM2 and PPM1D (11,12).

Expression of TP53 responsive genes can further be influenced by expression of the TP53 family members TP63 and TP73 and recent evidence suggests that all three family members can in certain contexts act as tumour suppressors or oncogenes dependent on the expression of other family members and their isoforms (reviewed in (1)). This is likely due to the transcriptional complexity exhibited by all three family members, which encode multiple isoforms as a result of alternative splicing and alternate promoter usage (2).

Unlike TP53 the role of TP63 in cancer is less clear. It is mutated in ∼7% of squamous cell carcinomas (13) and overexpressed/amplified in the majority of squamous cell carcinomas. However, expression is lost in other epithelial cancers such as breast, prostate and bladder tumours (14,15,16). Recent data suggests that ΔNTP63α represents a proto-oncogene, whereas TP63 behaves as a haplo-insufficient tumour suppressor and that these latter functions can be de-regulated by certain TP53 mutations (17–19).

The pervading model for the functional interplay between TP53 and TP63 is based largely on a relatively small number of canonical p53 activated anti-proliferative and pro-apoptotic target genes. The ΔNTP63α isoform opposes TP53- and TP73-mediated activation of anti-proliferative and pro-apoptotic target to promote proliferation and survival (20). For instance, in response to DNA damage ΔNTP63α can oppose TP53-mediated activation of anti-proliferative and pro-apoptotic target genes including CDKN1A, BAX and FAS (21). However, there is increasing evidence to suggest that TP53 can play both direct and indirect roles in the negative regulation of gene repression in response to genotoxic stress (22). Furthermore, recent studies in mice suggest that the transcriptional activation function of TP53 is at least in part dispensable for its tumour suppressive functions (23), underlining the importance of characterizing the genome-wide interplay that exists between these transcription factors.

The extent of this interplay is highlighted by recent studies from our laboratory and others, identifying dual roles for ΔNTP63α in the regenerating epidermis opposing TP53 activity to maintain proliferative capacity (24,25), whilst also being required for TP53 independent growth and differentiation (25). In addition, comparison of our recent TP63 ChIP-seq (26) analysis of genome-wide TP63 binding sites in primary human keratinocytes with TP53 ChIP-seq from disparate cell types (27–29) revealed more than 1000 overlapping binding sites.

Here we report the first genome-wide analysis of TP53 and TP63 binding sites in human keratinocytes. Using integrative analyses, we reveal that in response to genotoxic stress, changes in TP53 and TP63 bound cistromes result in induction or repression of a large network of target genes, the dynamics of which depends on the nature of the genotoxic stress.

Collectively, through direct effects of TP53 binding or indirect inactivation of TP63 this results in global repression of cell cycle and DNA damage repair genes concomitant with activation of anti-proliferative and pro-apoptotic canonical target genes. Importantly, our analyses reveal a role for TP63 in the constitutive maintenance of DNA repair genes, highlighting the importance of defining the TP53 /TP63 network in order to better understand the biological implication of de-regulation which frequently occurs in cancer.

## MATERIALS AND METHODS

### Cell culture

Primary neonatal Human Foreskin Keratinocytes (HFKs) were isolated as described previously (30) and cultured in Epilife supplemented with HKGS (GIBCO). HFKs were transfected with 50 nM siRNA using Lipofectamine RNAimax transfection agent (Invitrogen) according to manufacturer's instructions with siRNA-targeting total TP63, TP53 or a scrambled control. Cells were passaged 24 h post-transfection and various drug treatments conducted 48 h post-transfection and harvested after a further 24 h treatment (72 h post-transfection). Cell lines stably expression shRNA targeting TP53 or a scrambled control were generated as previously described (30).

### Chromatin immuno-precipitation, PCR and ChIP-seq

Chromatin immuno-precipitation was carried out based on Schmidt *et al.* (31–33) with the following amendments. Cells were cross-linked by addition of formaldehyde to a final concentration of 1% and incubated for 10 min at room temperature. Cross-linking was stopped by addition of glycine to a final concentration of 0.125 M washed and washed twice with ice-cold phosphate buffered saline (PBS). Nuclei were isolated as described in Schmidt *et al.* and equivalent of 1 × 10^6^ nuclei/ml of lysis buffer sonicated for 30 cycles (30 s on 30 s off, high power) in a Diagenode Bioruptor. Resulting chromatin supplemented with complete protease inhibitor cocktail (Roche) was incubated with 50 μl of protein-G Dynabeads (Invitrogen) pre-blocked and bound with 5 μg monoclonal antibodies (Santa Cruz; TP63-4A4; TP53-DO1) and incubated for 16–18 h at 4°C. ChIPs were washed five times with 1 ml Radioimmunoprecipitation assay buffer (RIPA) buffer and once with 1 ml TE buffer, eluted incubated overnight at 65°C to reverse cross-links, digested with RNAseA (Ambion) and proteinase K (Invitrogen) and purified previously described and libraries prepared for sequencing as previously described (26).

### Data analysis

Fastq files were generated with Illumina pipeline software (CASAVA 1.8.1 using the default chastity base call thresholds) and subsequently filtered to remove polymerase chain reaction (PCR) duplicates. Reads were mapped to the GRCh37/hg19 reference genome using Burrows Wheeler Alignment (BWA) allowing for gapped alignment and maximum five alignment locations for each read.

MACS peak calling algorithm (version 1.4; *P* = 1e−5, shiftsize = 100) (34), used to call peaks comparing to input. Resulting peaks were curated to remove Encode ‘dark regions’ (Encode Consensus Signal Artifact Regions) and spurious peaks (>1000 reads, mostly in centromeric regions) (35). See Supplementary file 7 for library characteristics. Results have been submitted to NCBI Gene Expression Omnibus (36) accession number GSE56640.

**Figure 1.**
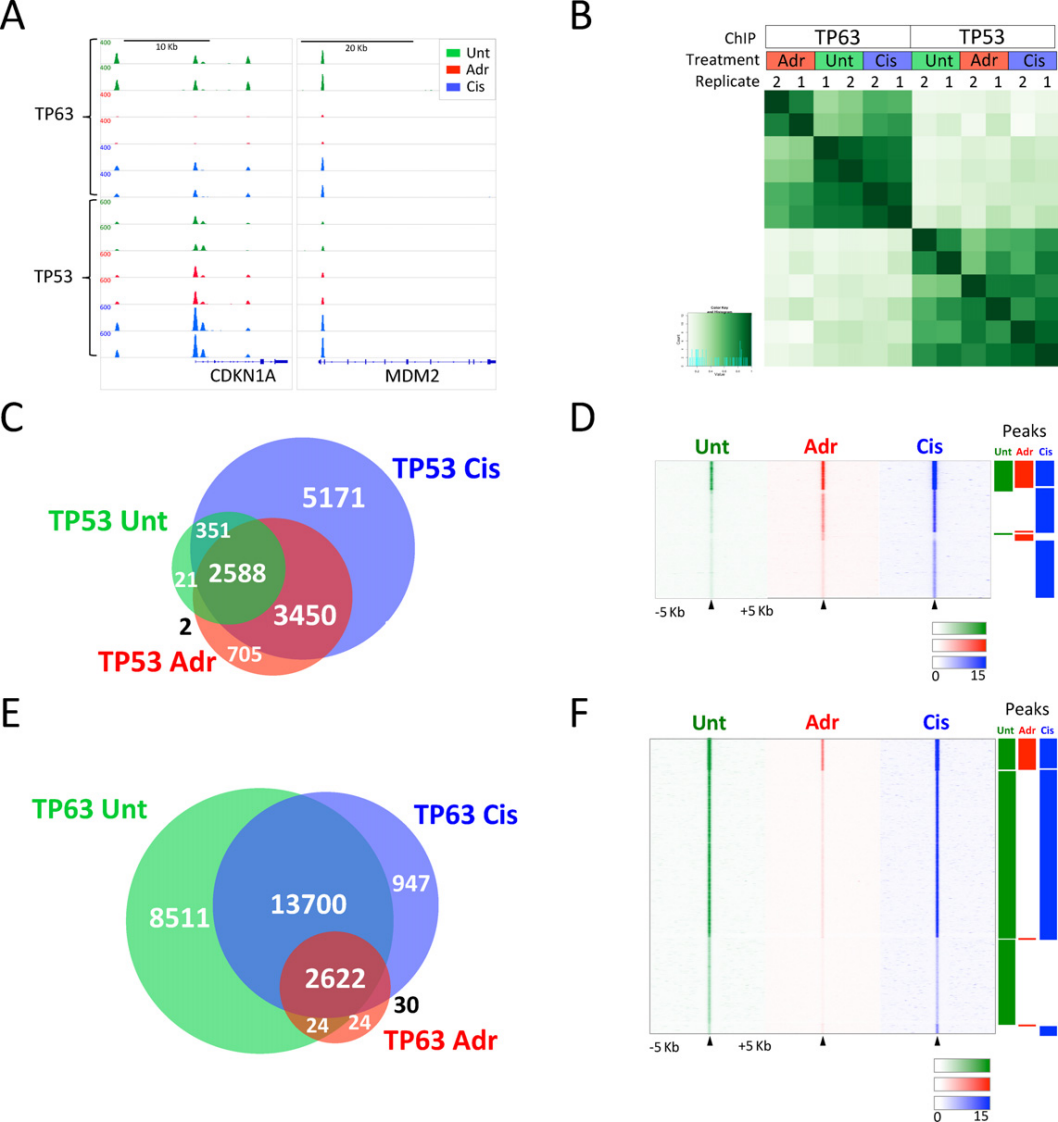
ChIP-seq analysis of TP53 and TP63 in untreated, adriamycin and cisplatin treated keratinocytes. (**A**) Normalized ChIP-seq biological replicate binding profiles around canonical TP53/TP63 target genes CDKN1A and MDM2, in the presence and absence of genotoxic agents adriamycin (350 nM) and cisplatin (25 μM). (**B**) Clustering of TP53 and TP63 ChIP-seq samples based on binding strength to sites identified using MACS in more than one sample compared using the Diffbind bioconductor package. (**C**) Venn diagram illustrating overlap of consensus TP53 peaksets present in both biological replicates. Where binding sites overlap with more than one site in other treatment sites are merged in Venn diagram. (**D**) Heatmap of individual TP53 binding sites, centred on peak maximum ± 5 kb. Clustered based on subsets of peaks identified in (C) as illustrated by bars. (**E**) Venn diagram illustrating overlap of consensus TP63 peaksets present in both biological replicates. (F) Heatmap of individual TP63 binding sites, centred on peak maximum ± 5 kb. Clustered based on subsets of peaks identified in (E) as illustrated by bars.

Resulting peak .bed files and .bam read files were used as input for DiffBind (25,37) to derive consensus peaksets extracted for each ChIP-seq factor/treatment combination (Figure 1C and E) and to generate heatmaps. Differential binding analysis was carried out with Diffbind according to manual using EDGER with libraries normalized to total library size.

For visualization IGVtools (38) from command line was used to generate .wig files, which were subsequently normalized (per 10 million reads) using wigreader (https://github.com/rgejman/wigreader) and visualized using the Integrative Genomics Viewer (38). Sitepro tool within Cis-regulatory Element Annotation System (CEAS) (39) was used to generate intensity profiles from normalized .wig files and outputs visualized within R environment (www.r-project.org).

Read counts across binding sites were generated using custom scripts (G. Brown, personal communication) and heatmaps visualized using Java TreeView (40).

To generate *de novo* motifs, Meme-ChIP (41) analysis was conducted on 500 bp sequences surrounding peak centre for peaksets (5000 randomly selected peaks if number greater). Conservation plots for the 3 kb regions surrounding the 7574 TP63 peaks was generated by plotting PhastCons for vertebrates using the conservation plot tool from CEAS (39) within the Cistrome (42) galaxy environment.

To correlate TP63 and TP53 peaks with potential target genes, we chose to annotate each peak to any gene within 25 kb of the identified peak. This was achieved by overlapping peaks with Refseq genes, transcription start sites (TSS) or termination sites extended by 25 kb downloaded from the University California Santa Cruz (UCSC) browser (43,44) within the Galaxy environment (45,46). In addition, peaks were mapped to genomic features using the CEAS tool from command line (27,47)

Seqminer (Version 1.3) (48) was used to extract histone modification data from Encode data for normal human epidermal keratinocytes (NHEKs) (49,50) and K-means clustering (raw) of this data carried out for respective sets of binding sites.

Gene ontology analysis was conducted using DAVID (32) and terms summarized using REVIGO (31).

### RNA extraction and quantitative RT-PCR

RNA extraction was carried out using Trizol (Invitrogen) according to the manufacturer's instructions. For quantitative RT-PCR, RNA was reverse transcribed to cDNA using the transcriptor high-fidelity cDNA synthesis kit (Roche), with random hexamers according to the manufacturer's instructions. Amplification of PCR products was monitored using Lightcycler 480 SYBR Green I Master (Roche) according to the manufacturer's instructions and fluorescence monitored on a Roche 480 Lightcycler and melting curve analysis also performed. In brief, cDNA samples were diluted 1:50 and quantified compared to a standard dilution series using the absolute relative quantitation method. The cycling conditions were as follows: initial denaturation 95°C for 10 min 45 cycles of 95°C, 15 s; 58°C, 15 s; 72°C, 20 s. Expression levels were assessed in triplicate, normalized to RPLPO and 18S control levels (for primer sequences see Supplementary file 8). Primers for TP53 and TP63 isoforms are previously published (25).

### Microarray analysis

For microarray analysis RNA was further purified using RNAeasy columns (Qiagen) according to manufacturer's instructions, before submission to Almac Diagnostics for analysis on the Affymetrix Exon ST 1.0 array platform.

Resulting .cel files were imported into Altanalyze software (43) and analysed using default settings. Gene expression was summarized at an individual gene level for ‘constitutive’ exons (probe sets aligning to those exons regions most common amongst all transcripts) filtering for probes with DABG *P* < 0.05 or expression <1. Fold change and log_2_ fold change for all samples was then calculated by comparing each sample relative to the mean of the no siRNA and scrambled untreated controls. Results have been submitted to NCBI Gene Expression Omnibus (36) accession number GSE56640.

For head and neck squamous cell carcinoma (HNSCC) array analysis raw .cel files were downloaded for Thurlow *et al.* from MIAME-VICE (http://bioinformatics.picr.man.ac.uk/vice/Welcome.vice) (51) and for Pyeon *et al.* (52) from NCBI Gene Expression Omnibus (GSE8791). Raw data was imported with RMA normalization into Partek Genomics Suite (Partek^®^ software. Copyright, Partek Inc., St Louis, MO, USA). Batch effects were removed and differential gene expression comparing tumour with normal was determined using a three-way ANOVA model incorporating tumour/normal, site and HPV status (Thurlow *et al.* p16 staining, Pyeon *et al.* HPV microarray hybridization). Pyeon *et al.* included 14 normals and 40 HNSCC cases. Data from Thurlow *et al.* was pre-filtered to remove duplicates and retain only samples from only oral cavity, oropharynx and larynx (75 total arrays including 13 normals).

Hypergeometric distribution was used to calculate significance of genelist overlaps (53), which predicts the probability of the overlap of the two gene lists, given the length of the two lists and the number of genes that could have been present on both lists (R-code available on request).

### Western blot analysis

Western blots were carried out as previously described (25). Primary antibodies used were Santa Cruz monoclonal anti-TP63 (4A4), TP53 (DO1), CDKN1A (C19), FANCD2 (FL17), polyclonal TP53-pS15 (Cell Signalling), TP53-pS46 (Abcam), BRCA1 (Sigma prestige), beta-actin (Sigma), RRM2B (Abcam). Secondary antibodies used in this study were goat anti-mouse and rabbit-HRP (Santa Cruz). Luminescence was revealed by incubation with Western Lightning ECL (Perkin-Elmer) and signal detected on an Alpha Innotech FluorChem™ SP imaging system.

### Indirect immunofluorescence

Cells were transfected with siRNAs as above and plated onto coverslips 24 h post-transfection, after a further 24 h coverslips were exposed to 2 Gy ionizing radiation (IR). Cells were fixed with 4% paraformaldehyde at the indicated time points, permeabilized in 0.1% Triton X-100/PBS and blocked in 10% foetal bovine serum/PBS. Cells were stained for γ-H2AX (Millipore) and 53BP1 (Millipore), anti-TP63 (4A4, Santa Cruz), TP53 (DO1, Santa Cruz), primary antibodies overnight at 4°C, washed and stained with goat anti-rabbit Alexafluor 488 or anti-mouse 568 Fab′2 fragment secondary antibodies (LifeTechnologies) washed and mounted in Prolong Gold (LifeTechnologies) containing 4′,6-diamidino-2-phenylindole (DAPI) to visualize nuclei. Cells were visualized and foci counted using a Nikon Eclipse Ti fluorescence microscope, using a ×60 objective.

### FACS analysis

Cells were fixed and stained for PI as previously described (54) and analysed for PI content on a Becton Dickinson LSR flow cytometer.

## RESULTS

### Genome-wide characterization of TP53 and TP63 cistromes in response to genotoxic stress

To investigate the genome-wide interplay between TP53 and TP63 binding sites, we used primary neonatal HFKs as an exemplar of epithelial cells and which express high levels of TP63 and wild-type TP53. HFKs express high amount of ΔNTP63, predominantly the α C-terminal variant (25), but undetectable levels of the TA isoforms, while they express high levels of full length TP53 (Supplemental Figure S1A). Genome-wide TP53 and TP63 binding sites were mapped using ChIP-seq validated antibodies, which recognize all isoforms of TP63 and TP53 (Supplementary Figure S1A) (26,29,55,56) in two independent sets of primary HFKs, in the presence and absence of genotoxic agents cisplatin and adriamycin for 24 h. This timeframe resulted in maximal TP53 stabilization (Supplementary Figure S1B), binding and activation of target genes such as *CDKN1A* (Supplementary Figure S1C and D), but with only modest effects on cell cycle (Supplementary Figure 1E). Activation is through stabilization of canonical full-length TP53 isoform TP53α (Supplementary Figure S1A, B and G) and strong serine-15 phosphorylation and to a lesser extent on serine-46 (Supplementary Figure S1B). This TP53 activation is concomitant with reduced levels of TP63 protein and mRNA (Supplementary Figure S1A, B and F), which is greater at the protein level following adriamycin treatment (Supplementary Figure S1A) than in cisplatin treated cells. Visual inspection of normalized binding profiles around the canonical CDKN1A and MDM2 promoters (Figure 1A) and clustering based on global occupancy and intensity of binding demonstrates the reproducibility between replicates (Figure 1B).

To generate robust consensus peaksets, we considered only peaks detected in both replicates of each ChIP/treatment combination using the MACS peak-calling algorithm (34) (Supplementary Figure S2). Based on these criteria, we cumulatively identified a total of 12 287 TP53 sites (Figure 1C and D) (merged from 12 378 total) and 25 858 TP63 sites (Figure 1E and F). Both TP53 and TP63 sites are highly conserved and enriched for a similar *de novo* TP53 family motif broadly focused around Refseq TSS (Supplementary Figure S3). This enrichment of TP63 and TP53 around TSS is further supported by more detailed analysis of genomic location of subsets of sites identified in each of the treatment antibody combinations measured (Supplementary Figure S3D and E). This indicates that constitutive TP63 and TP53 sites in cells treated with genotoxic agents are highly enriched in 5′UTRs, bi- and uni-directional promoters.

Comparison of the subsets of peaks identified in different treatment conditions reveals 2962 TP53 sites constitutively bound by TP53 in untreated HFKs. The number identified increases concomitant with TP53 stabilization to 6745 sites upon adriamycin treatment and to 11 560 upon cisplatin treatment (Figure 1C and D and Supplementary file 2). In contrast, a large proportion of TP63 binding events are detected in untreated cells (Figure 1E and F), the majority of which are lost upon adriamycin treatment (Figure 1E and F and Supplementary file 2), concomitant with a decrease in TP63 levels (Supplementary Figure S1A). Somewhat, surprisingly, upon cisplatin treatment a large proportion of TP63 peaks are still detected (Figure 1E and F), implying that despite a decrease in TP63 protein levels and binding, ‘occupancy’ is affected to a lesser extent than adriamycin treatment.

Taken together, this suggests that differential dynamics of TP53 and TP63 binding depend on nature and intensity of genotoxic stress.

### Comparison of TP53 and TP63 cistromes reveals overlapping and distinct binding sites

**Figure 2.**
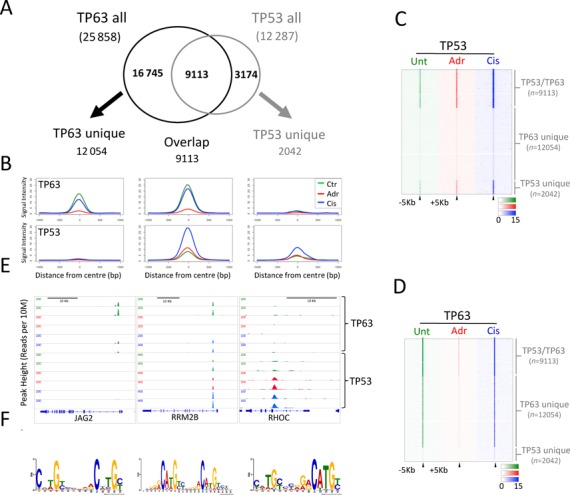
TP53 and TP63 bind to overlapping and distinct subsets of binding sites. (**A**) Venn diagram illustrating the overall number of sites bound by both TP53 and TP63 in any of the conditions tested. TP53 unique sites (2042) were identified as those present in both replicates of any condition, but not present in any TP63 sample and the converse true for the 12054 TP63 unique sites. (**B**) Plot of normalized binding intensity across the TP63 unique, overlapping and TP53 unique sites for TP63 and TP53 across the three treatment conditions. (C and D) Heatmaps of individual TP53 (**C**) and TP63 (**D**) binding sites, centred on peak maximum ± 5 kb. Clustered based on subsets of peaks identified as overlapping, TP53 unique and TP63 unique sites. (**E**) Illustrative examples of ChIP-seq results for TP63 unique, overlapping and TP53 unique sites. (**F**) *De novo* motifs identified from analysis of TP63 unique, overlapping and TP53 unique sites.

To determine the extent of overlap between TP53 and TP63 cistromes, we first overlapped our pooled consensus TP53/TP63 binding sites (Figure 2A). This indicates that the majority of TP53 sites (9113) are also bound by TP63 in at least one of the conditions measured, whereas the converse is not true for TP63, where more than half the sites are not bound by TP53 (Figure 2A). To robustly define TP53 and TP63 only bound sites, peaks present in any individual sample were further subtracted from the non-overlapping 16 745 TP63 or 3174 TP53 consensus peaks to identify 12 054 unique peaks bound only by TP63 and 2042 bound only by TP53 (Figure 2A). This is illustrated by global (Figure 2B) and individual (Figure 2C and D) signal intensity profiles and by sites associated with the *JAG2*, *RHOC* and *RRM2B* genes, respectively (Figure 2E). Examination of the average strength indicates that binding of both TP53 and TP63 is highest for those sites, which can be bound by both TP53 and TP63 (Figure 2B). The robustness of the peaksets is supported by *de novo* motif analysis, which identifies enrichment for a TP53/TP63-like binding motif in the majority of each of these subsets of binding sites. Specifically, a canonical TP53/TP63 motif is detected centrally, within 76% of TP53/TP63 overlapping peaks (Figure 2F and Supplementary Figure S4). A similar but distinct *de novo* motif is present in 74% of TP63 unique peaks (Figure 2F and Supplementary Figure S4), whereas a more degenerate motif a highly conserved half site is associated with 68% of TP53 unique sites (Figure 2F and Supplementary Figure S4). Further analysis revealed that one TP53 half site motif (CxxG) is enriched for in 83, 87 and 78% of the overlapping and TP63 and TP53 unique peaksets, respectively (Supplementary Figure S4). The more highly conserved binding motifs observed in TP63 and TP63/TP53 overlapping sites correlates with greater conservation than TP53 only sites (Supplementary Figure S5A). While all sites localize broadly to areas surrounding Refseq TSS (Supplementary Figure S5B), the TP53 only sites are more frequently located in proximal promoter regions associated with high histone H3K4Me3 in cycling NHEKs (ChIP-seq data available from the ENCODE project (35)), whereas TP63 only sites are more frequently associated with enhancer-like regions associated with high histone H3K4me1 (Supplementary Figure S6A) (47). Furthermore, stratification of TP53 and TP63 based on nearest Refseq TSS reveals that as anticipated TP53 and TP63 distal sites (5–25 and >5 kb) are more frequently associated enhancer-like marks in NHEK cells, whereas more proximal binding events (<5 kb) are associated more frequently as expected with promoter associated histone marks (Supplementary Figure S6B).

### Differential binding analysis reveals differences in TP53/TP63 binding dynamics in response to different genotoxic agents

**Figure 3.**
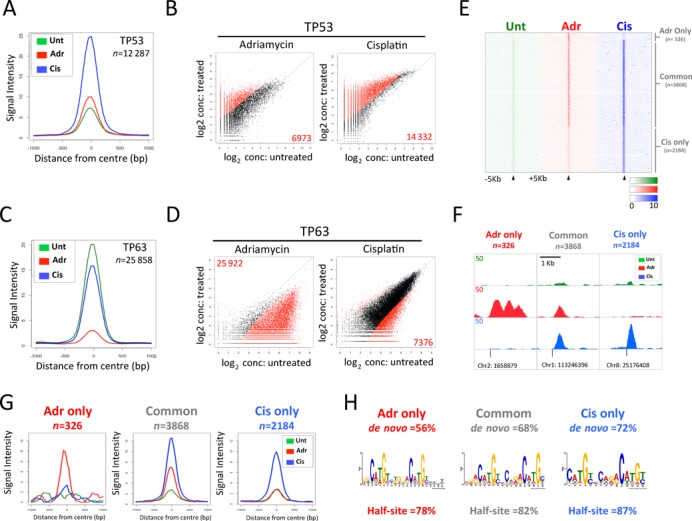
Characterization of differential TP53/TP63 binding dynamics in response to adriamycin or cisplatin treatment. (**A**) Normalized TP53 binding intensity across all TP53 in untreated HFKs or following adriamycin or cisplatin treatment. (**B**) Differential TP53 binding site analysis. log_2_ concentration plots of pairwise comparisons of mean normalized read count. Significant changes in bound sites indicated in red (FDR < 0.1). (**C**) Normalized TP63 binding intensity across all TP53 in untreated HFKs or following adriamycin or cisplatin treatment. (**D**) Differential TP63 binding site analysis. log_2_ concentration plots of pairwise comparisons of mean normalized read count. Significant changes in bound sites indicated in red (FDR < 0.1). (**E**) Illustrative examples of ChIP-seq results for TP53 adriamycin only, cisplatin only and overlapping sites induced upon genotoxic stress. (**F**) Normalized TP53 binding intensity across adriamycin only, cisplatin only and overlapping TP53 sites induced upon genotoxic stress. (**G**) Plot of normalized binding intensity across all of the TP63 and TP53 sites for each of the three treatment condition. (**H**) Summary of *de novo* motif analysis of adriamycin only, cisplatin only and overlapping sites damage-induced TP53 sites.

Comparison of normalized TP53 signal intensity across all 12 287 TP53 sites reveals that, similar to occupancy-based analyses, TP53 binding is increased to the greatest extent following cisplatin treatment (Figure 3A). The significance of these differences is supported by differential binding analysis (37) which identifies 14 332 sites exhibiting a significant increase in TP53 binding following cisplatin treatment compared with untreated cells (FDR < 0.1). This is in comparison to a significant increase of only 6973 sites upon adriamycin treatment (Figure 3B). Taken together, these results indicate that there is a greater increase in the number of sites bound by TP53, and with greater intensity, after cisplatin treatment compared with adriamycin treatment. The converse is true for TP63, where global TP63 binding is decreased more dramatically upon adriamycin treatment than cisplatin (Figure 3C). This is supported by differential binding analysis, which reveals that TP63 binding is significantly decreased by >25 000 sites when comparing adriamycin treatment with untreated cells and only 7396 TP63 sites significantly reduced upon cisplatin treatment (Figure 3D).

These quantitative analyses, support differing modes of regulation utilized by TP53 in response to adriamycin and cisplatin treatment. By subtractive analysis, we can in fact discern peaks induced specifically by cisplatin (2184), adriamycin (326) or 3868 overlapping damage-induced TP53 binding events each of which is significantly enriched for *de novo* TP53-like binding motifs or half sites (Figure 3E–H). This highlights that not only do these different types of stress elicit different global effects on TP53 dynamics, but also that there are damage-induced sites, which are specific to each type of DNA lesion/agent. In contrast, similar subtractive analysis reveals that only 182 and 10 TP63-specific new binding events are detected subsequent to cisplatin and adriamycin treatments, respectively (data not shown) re-enforcing the fact that the vast majority of TP63 sites are detected in cycling cells.

### Characterization of global effects on transcription mediated by TP53 and TP63 in response to genotoxic stress

To identify genes whose expression could be influenced by the TP53 and TP63 cistromes, we first annotated peaks to REFSEQ genes identifying 9087 and 13 403 genes within 25 kb of a TP53 or TP63 binding event respectively regardless of treatment (Supplementary Figure S7, Supplementary file 1 and Supplementary file 3). Therefore, TP53 and TP63 have the potential to directly influence expression of a large proportion of the genome. This threshold was initially chosen since data from our laboratory and others has revealed potential for regulatory events to occur in more distal regions for TP63, TP53 and other factors such as estrogen receptor (26,55,57).

To gain a global perspective of the downstream effects of these binding events we integrated binding data with mRNA expression data generated by exon-array profiling, carried out concomitantly with one of the ChIP-seq replicates. Specifically, the same batch of HFKs cells were transfected with siRNA targeting TP53, TP63, scrambled control or untreated control. These cells were then treated in parallel with the cells used for ChIP-seq with cisplatin or adriamycin for 24 h and RNA extracted and submitted for Affymetrix Exon Array analysis. Results were analysed using the Altanalyze software package (43) at an individual gene level by combining results for constitutively spliced exons. Fold change in response to treatment was then calculated by comparing adriamycin or cisplatin treatment relative to the mean of the no siRNA and scrambled untreated.

**Figure 4.**
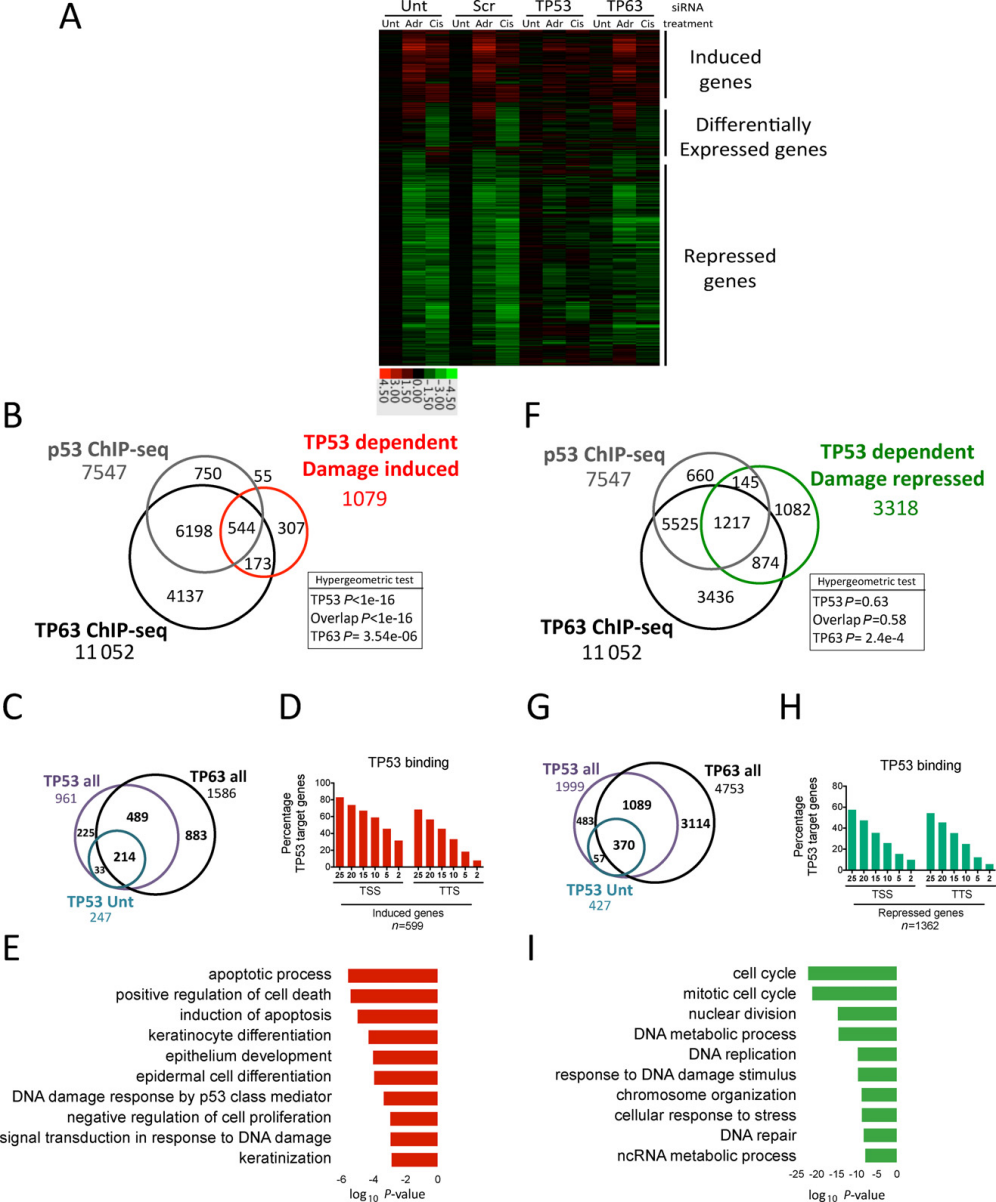
Integration of ChIP-seq and microarray data reveals complex interplay between TP53 and TP63. (**A**) Heatmap of microarray data showing TP53 and TP53/TP63 and TP63 bound genes. (**B**) Comparison of genes induced in a TP53-dependent manner upon either adriamycin or cisplatin treatment with those within TP53 and TP63 binding sites identifies 772 potential target genes. (**C**) Analysis of TP53 and TP63 sites within 25 kb of 772 induced genes from (B) superimposed with genes associated with TP53 binding in untreated cells. (**D**) Histogram comparing distance of TP53 sites from transcription start sites (TSS) and transcription termination sites (TTS) of 772 induced genes. (**E**) Gene ontology analysis of 772 induced genes. (**F**) Comparison of genes repressed in a TP53-dependent manner upon either adriamycin or cisplatin treatment with those within TP53 and TP63 binding sites identifies 2236 potential target genes. (**G**) Analysis of TP53 and TP63 sites within 25 kb of 2236 repressed genes from (F) superimposed with genes associated with TP53 binding in untreated cells. (**H**) Histogram comparing distance of TP53 sites from TSS and transcription termination sites of 772 induced genes. (**I**) Gene ontology analysis of 2236 repressed genes identified.

Using this strategy, we identify 1563 and 1153 genes induced at least 1.7-fold by adriamycin or cisplatin treatment, respectively (Supplementary Figure S8A and Supplementary file 4). In both treatments, induction of a significant proportion of these genes (700 adriamycin and 463 cisplatin) was prevented in TP53 siRNA depleted cells (1.5-fold change in opposite direction) (Supplementary Figure S8), indicating that this induction is TP53-dependent. A highly significant proportion of these genes (Figure 4A and B and Supplementary file 5) (599, hypergeometric *P* < 1e−16) are also within 25 kb of 961 TP53 binding sites, potentially representing direct TP53-induced target genes (Figure 4B and C), many of which, such as *CDKN1A*, are associated with multiple TP53 sites.

A significant subset (219) of these TP53-induced genes are within 25 kb of constitutive TP53 binding sites in untreated cells, 123 of these sites are also associated with at least one additional DNA-damage-induced TP53 site (Figure 4C and Supplementary Figure S9A), while an additional 380 induced genes are associated with sites only detected upon damage. These 599 damage-induced genes are enriched, for TSS/promoter proximal TP53 binding events as opposed to 3-prime binding at TTS (Figure 4D and Supplementary Figures S3D and E and S8B). Together, this implies complex modes of activation of TP53 target genes, through increased binding or activation of sites already bound by TP53 and/or induction of TP53 binding to novel sites. The intricacy of these regulatory events is highlighted by the presence of multiple TP53/TP63 binding sites at various distances relative to the TSS of both induced and repressed genes (Supplementary Figure S9C and D).

These analyses are complicated by the presence of TP63 on the majority of TP53 sites (Figure 4C), which potentially influences TP53 activity through transcriptional inhibition and/or competitive binding. As such, we also observe significant enrichment for pooled TP63 binding sites within 25 kb of induced genes (Figure 4B and Supplementary Figure S8A, Supplementary file 1 and Supplementary file 4). Most of these genes are associated with sites bound by both TP53 and TP63 in at least one of the conditions measured (Figure 4C and Supplementary Figure S9B), indicating that these represent canonical target genes whose activation is induced by TP53 but opposed in cycling cells by TP63 binding.

Interestingly, the number of genes induced by cisplatin (195) associated with TP53 binding was significantly less than that induced by adriamycin (457), (Supplementary Figure S7), perhaps reflecting the differing binding dynamics and/or biological effects of the doses and time points chosen (Supplementary Figure S1). Cumulatively, these 772 TP53-damage-induced targets associated with TP53 and/or TP63 binding (Figure 4B) are enriched for genes involved in apoptosis, cell cycle arrest, DNA damage and stress response (Figure 4E and Supplementary Figure S9A).

Significantly, using the same selection criteria, both genotoxic agents resulted in repression of a greater number of genes in a TP53-dependent manner, a large proportion of which (1362) were also within 25 kb of a TP53 site, considering both genotoxic agents (Figure 4F and Supplementary Figure S8A, Supplementary file 4). In contrast to induced genes, TP53 sites associated with repressed genes are not enriched in promoter proximal regions (Figure 4H), rather they are associated with *de novo* damage-dependent events at more distal binding sites (Supplementary Figure S9E). This was particularly apparent for cisplatin treatment, which resulted in TP53-dependent down-regulation of a larger total number of genes (1160) than associated with adriamycin treatment (557) (Supplementary Figure S7). Like induced genes, the majority of TP53 sites within 25 kb of repressed genes are also bound by TP63 in one of the conditions measured (Figure 4G). Importantly, a highly significant number of TP63-specific binding events occur within 25 kb of TP53-dependent repressed genes (Figure 4G and Supplementary Figure S9F), suggesting that that TP53 can negatively regulate expression of a subset of genes through affecting TP63 transcriptional activity, DNA binding, protein levels or a combination thereof. In fact, expression array data indicates that TP63 depletion alone is sufficient to result in down-regulation of a subset of these genes that are repressed after DNA damage in a TP53-dependent matter (Figure 4A and Supplementary Figure S10). Collectively, the 2236 (Figure 4F) genes we identify as being repressed in a TP53-dependent manner and associated with TP53/TP63, TP63 only or TP53 only binding sites are highly enriched for genes involved in cell cycle progression, DNA metabolism and repair (Figure 4I and Supplementary Figure S10).

Our global analyses indicate that TP53 can positively and negatively influence the expression of a large number of genes in response to DNA damage and that TP63 potentially plays an important role in regulating these events. Specifically, our results suggest that activation events are likely mediated directly by TP53, through a combination of increased binding, activation of pre-bound protein, or loss of repression mediated by TP63.

Furthermore, analysis of all TP53 and TP63 sites associated with induced genes indicates they have higher levels of H3K4me3 in NHEK cells (35), indicative of promoter-like elements (47), whereas sites associated with repressed genes have low H3K4me3 and high H3K4me1 levels, indicative of enhancer-like elements (Supplementary Figure S11). Therefore, TP53 potentially elicits its suppressive effect through modulation of enhancer activity, which is constitutively bound by TP63.

### TP53 and TP63 interplay in regulating DNA damage repair genes

**Figure 5.**
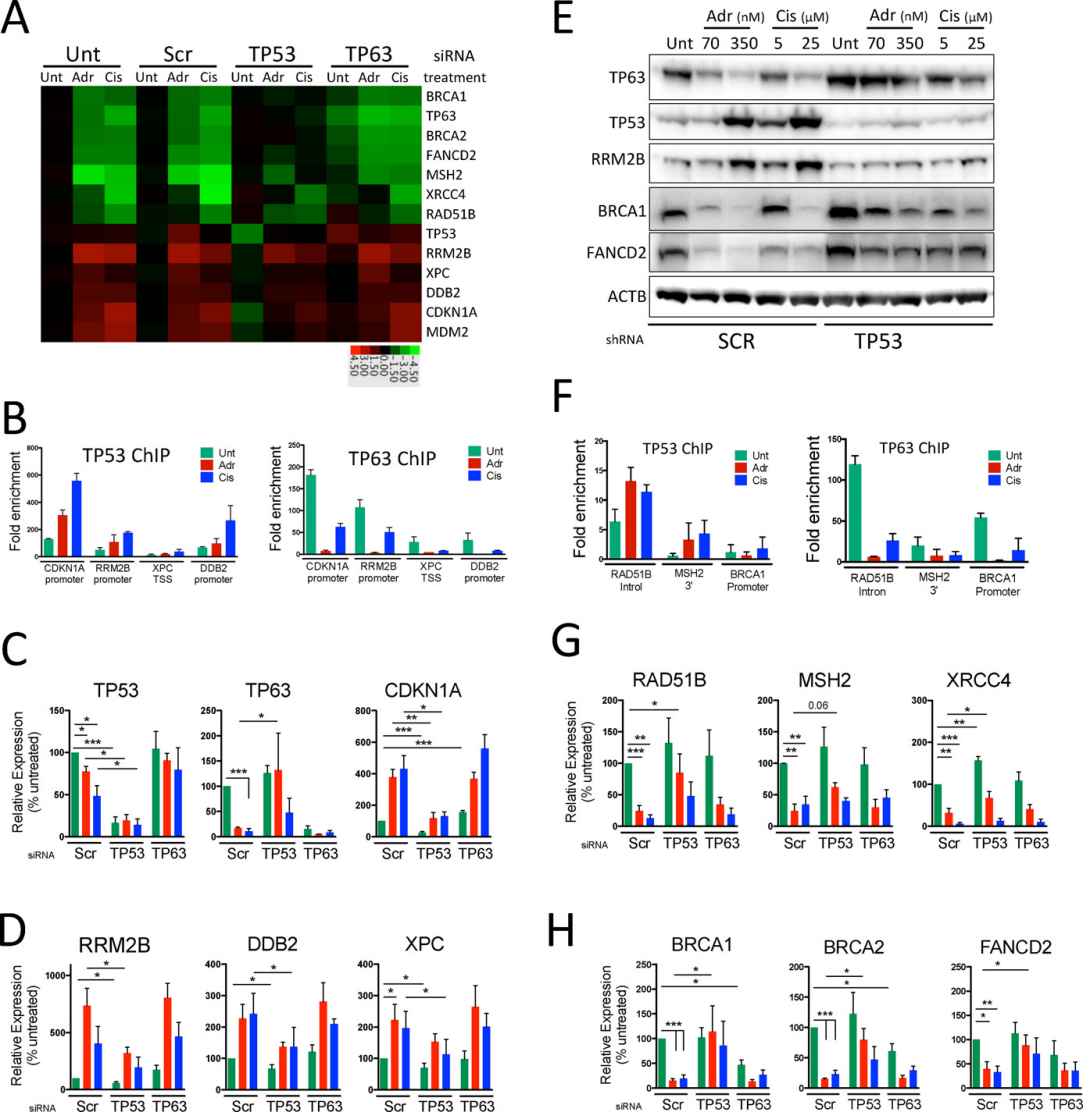
Characterization of effects of role of TP53 and TP63 in regulation of DNA repair genes. (**A**) Heatmap of microarray results for selected DNA damage repair genes. Data expressed as log_2_ fold change compared with average of untreated controls. (**B**) Quantification of TP53 and TP63 binding to regions associated with TP53-dependent induced repair genes XPC, DDB2, RRM2B and positive control CDKN1A by ChIP- followed by quantitative PCR (qChIP-PCR). (**C**) Confirmation by qRT-PCR of TP53 and TP63 depletion and effect on expression of CDKN1A positive control in adriamycin or cisplatin treated HFKs. (**D**) Quantitation by qRT-PCR of effects of adriamycin or cisplatin treatment on mRNA expression of TP53-dependent induced DNA repair genes RRM2B, DDB2 and XPC in TP53 and TP63 depleted HFKs. (**E**) Western blot analysis of TP53, TP63, RRM2B, BRCA1, FANCD2 and control CDKN1A protein expression in HFKs stably depleted for TP53 in response to treatment with increasing amounts of adriamycin (70 nM/350 nM) or cisplatin (5 μM/25 μM). (**F**) Quantification of TP53 and TP63 binding to regions associated with TP53 dependently repressed repair genes RAD51B, MSH2, BRCA1 by qChIP-PCR. (**G**) Quantitation by qRT-PCR of effects of adriamycin or cisplatin treatment on mRNA expression of TP53/TP63 bound TP53-dependent repressed DNA repair genes RAD51B, MSH2. (**H**) Quantitation by qRT-PCR of effects of adriamycin or cisplatin treatment on mRNA expression of TP63 only bound TP53-dependent repressed DNA repair genes BRCA1, BRCA2 and FANCD2. qChIP-PCR show mean ± SD. qRT-PCR shows mean ± SEM of at least three biological replicates. *P*-values calculated with Student's *t*-test. *P < 0.05; **P < 0.01; ***P < 0.001.

Our global findings suggest a complex interplay between TP53 and TP63 resulting in a number of potential modes of regulation, dependent on the individual gene and the type and intensity of genotoxic stress. We next sought to validate these different modes of co-regulation of specific target genes. Interestingly, gene ontology analyses, indicated significant enrichment for TP53-dependent repression and induction of genes, involved in DNA damage response (Figure 4E and I and Supplementary Figure S10). These included genes encoding proteins involved in homologous recombination (HR)/Fanconi Anemia (FA) pathway (RAD51B, BRCA1, FANCD2, BRCA2), Non-Homologous end Joining (e.g. XRCC4) and mismatch repair (e.g. MSH2) (Figure 5A). We also observed up-regulation of components of the nucleotide excision repair pathway (XPC, DDB2) and DNA damage responsive nucleotide biosynthesis (RRM2B), which have been previously identified as TP53 targets (58,59). We chose to validate a subset of these repair genes/proteins associated with different potential modes of regulation. Specifically, we examined: (i) induced genes (RRM2B, DDB2 and XPC) associated with both TP53 and TP63 binding; (ii) repressed genes associated with both TP53/TP63 sites (*RAD51B*, *MSH2* and *XRCC4*) and (iii) those with TP63 binding only within 25 kb (*BRCA1*, *BRCA2* and *FANCD2*) (Figure 5A).

Validation of these promoter proximal events by quantitative ChIP-PCR (qChIP-PCR) correlates with ChIP-seq results, with induced binding of TP53 to *RRM2B* and *DDB2* promoters and a weaker binding to the *XPC* promoter (Figure 5B). Interestingly, binding of TP63 to all of these TP53 bound sites was also observed in untreated cells (Figure 5B and Supplementary Figure S12A and B) and decreased, particularly upon adriamycin treatment (Figure 5B). To correlate binding events with effects on gene expression, we conducted RT-PCR (qRT-PCR) from primary HFKs transiently depleted for TP53 or TP63 in the presence or absence of adriamycin or cisplatin treatment and confirmed effects on CKDN1A expression as a positive control (Figure 5C). TP53 depletion results in attenuation of induction of DNA repair genes RRM2B, DDB2, XPC and the positive control CKDN1A (Figure 5C and D). These effects are titratable at both the mRNA and protein level (Figure 5E and Supplementary Figure S13). Depletion of TP63 alone is sufficient to increase levels of RRM2B and CDKN1A, implying that TP53 activity is repressed by TP63 in the absence of genotoxic stress (Figure 5C).

In contrast to induced genes, TP53 binding events associated with gene repression are more frequently new binding sites upon genotoxic stress (Supplementary Figure S9A and D) and are less strongly bound as measured by fold enrichment and peak height (*RAD51B*, *MSH2* and *XRCC4*) (Figure 5F and Supplementary Figure S12C–E, Supplementary file 1). Quantitative RT-PCR in transiently TP53 or TP63 depleted HFKs confirms that down-regulation of RAD51B, MSH2 and XRCC4 occur in a TP53-dependent manner in both transiently and stably TP53 depleted cells (Figure 5G and Figure S13C–E). Unlike, TP53-induced genes TP63 depletion alone did not effect mRNA expression of RAD51B, MSH2 or XRCC4 (Figure 5G), indicating that a genotoxic signal is required to activate these events.

Interestingly, TP63 mRNA and protein levels were both observed to be reduced in a TP53-dependent manner upon adriamycin and cisplatin treatment since TP53 depletion resulted in higher expression of TP63 (Figure 5C and Supplementary Figure S13). In support of an interaction between TP53 and TP63 expression, our global analyses revealed a significant enrichment for genes associated with TP63 only binding as being repressed in a TP53-dependent manner in response to genotoxic treatment (Figure 4F and Supplementary Figure S8A, Supplementary file 4). Interestingly, within the DNA repair associated subset we noticed a number of core effectors of homologous recombination (BRCA1, BRCA2 and FANCD2) that exemplified this interaction (Figure 5F and H and Supplementary Figure S12F–H). As predicted from our expression array results (Figure 5A), quantitative RT-PCR confirmed that not only are these genes repressed in a TP53-dependent manner in response to genotoxic stress, but that TP63 depletion alone was sufficient to significantly reduce expression of BRCA1 and BRCA2 in untreated cells (Figure 5H) suggesting that TP63 plays a role in maintaining expression of these genes.

### TP63 affects repair of double strand breaks in a TP53 independent manner

**Figure 6.**
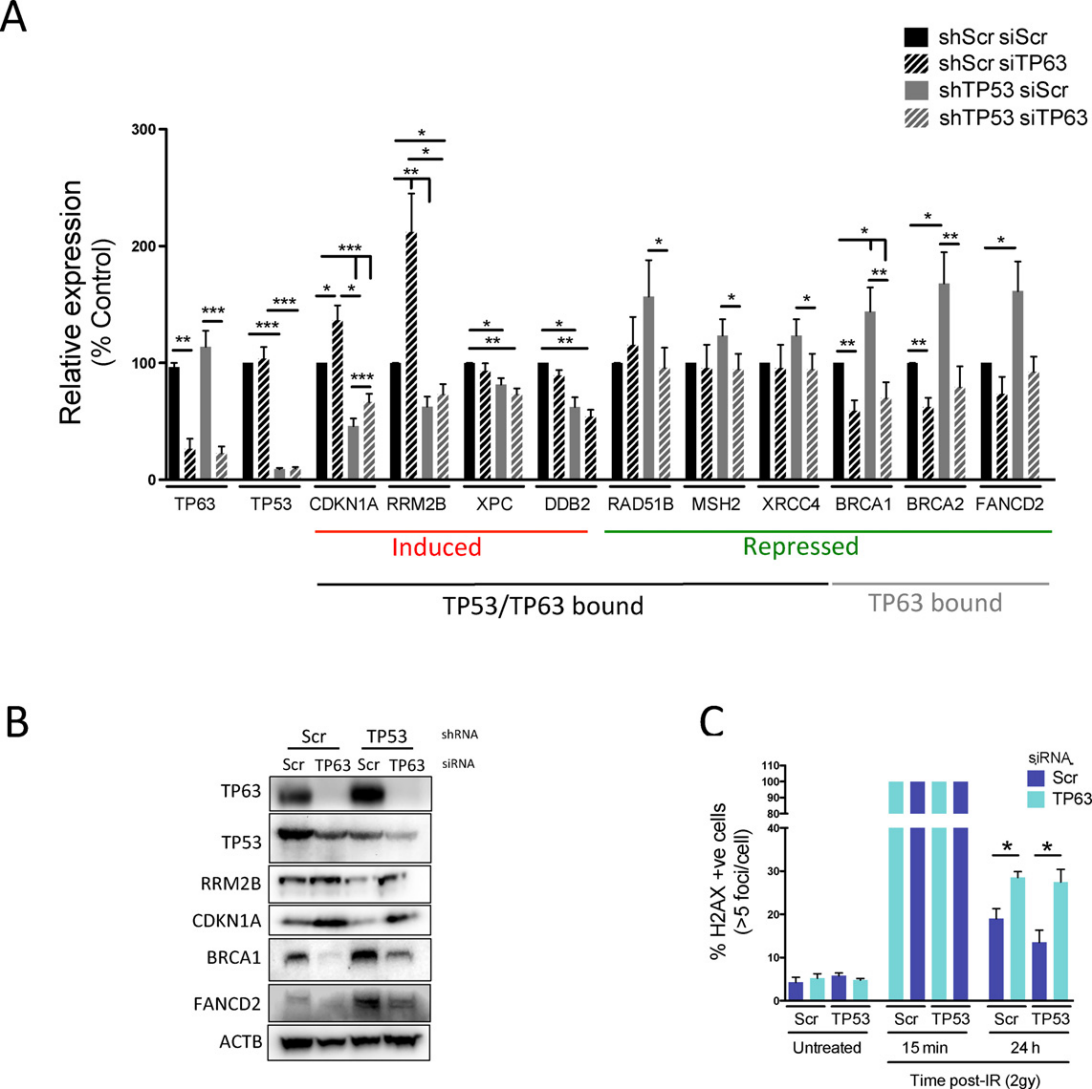
TP63 plays TP53-dependent and independent roles in basal expression of DNA repair genes. (**A**) Quantitation by qRT-PCR of effects of transient siRNA-mediated TP63 depletion on constitutive mRNA expression of DNA repair genes in stably TP53 depleted and scrambled control HFK lines. (**B**) Western blot analysis of effects of transient TP63 depletion on expression of RRM2B, CDKN1A, BRCA1 and FANCD2 in stably TP53 depleted and scrambled control HFK lines. (**C**) Analysis of effects of transient TP63 depletion on 53BP1/H2AX Foci resolution following treatment with 2 Gy ionizing radiation (IR) in stably TP53 depleted and scrambled control HFK lines. qRT-PCR shows mean ± SEM of at least three biological replicates. 53BP1/H2AX results represent the number of cells with >5 foci *n* >100 cells for three independent replicates. *P*-values calculated with Student's *t*-test. *P < 0.05; **P < 0.01; ***P < 0.001.

Our results suggest that TP53 can affect expression of DNA damage repair genes directly and indirectly through influencing TP63 levels and activity. This suggests that TP63 may play a role in maintaining constitutive expression of a subset of these genes, in particular those devoid of TP53 proximal binding events, whilst repressing activation of those genes constitutively bound by TP53. To test these hypotheses, we assessed constitutive expression levels of DNA damage repair genes upon TP63 depletion in HFK lines stably depleted of TP53 or scrambled control. Depletion of TP63 results in constitutive up-regulation of RRM2B and CDKN1A, which is attenuated in the shTP53 background (Figure 6A and B). This indicates that as for CDKN1A, TP63 actively represses the *RRM2B* promoter in the absence of genotoxic insult. This is not the case for all TP53-activated targets, since XPC and DDB2 are not activated upon TP63 depletion (Figure 6A) and stable TP53 depletion alone only has a minor effect, implying that a DNA damage signal is required to activate transcription of these targets. A similar complexity is observed for repressed genes, associated with TP53 and TP63 binding, namely *RAD51B*, *MSH2* and *XRCC4*, which are unaffected by TP63 depletion alone. However, we do observe a modest increase in expression of these genes in TP53 depleted cells, which is TP63-dependent (Figure 6A). Importantly, BRCA1, BRCA2 and FANCD2, which were associated with TP63 binding only, are significantly decreased upon TP63 depletion in both TP53 proficient and deficient cells (Figure 6A), implying that TP63 plays TP53 independent roles in their regulation.

If TP63 were involved in constitutively regulating DNA damage repair genes such as *BRCA1* and *BRCA2*, we would predict that there is a consequence for the cell when TP63 is depleted. To test this hypothesis, we treated TP63 depleted cells in the presence or absence of TP53 in order to control for TP53-dependent effects. These cells were then treated with ionizing radiation to induce DNA double strand breaks and their ability to resolve this damage monitored by quantification of phospho-gamma H2AX foci over time. No significant effect on basal number of foci was observed in the absence of IR, however, 24 h post-treatment, we observed significantly higher numbers of unresolved foci in TP63 depleted cells regardless of TP53 status (Figure 6C).

Taken together, the results suggest that in the absence of genotoxic insult TP63 plays a role in maintaining expression of genes important for DNA repair, in addition to its role in preventing spurious activation of TP53-mediated constitutive targets. In addition, we predicted genes that are repressed by TP53 would be up regulated in cancers that harbour a TP53 mutation, with high expression of TP63. Head and neck cancers have a high incidence of mutant TP53 (13,50) and high expression of TP63. Therefore, to determine the biological relevance of our results we interrogated two gene expression profiling datasets of head and neck cancers. This revealed that these cancers express significantly higher levels of genes (473 and 414) normally repressed by TP53 and that these up-regulated gene sets are enriched for genes involved in DNA repair and cell cycle progression (Supplementary Figures S14 and S5)

In summary, we have carried out ChIP-seq for TP53 and TP63 in primary human keratinocytes before and after DNA damage and integrated this with microarray data from the same cells. The data indicate that TP53 is bound to many fewer sites in cycling cells than TP63, but upon DNA damage TP63 is replaced by TP53 binding resulting in repression of genes involved in cell cycle, DNA repair and metabolism.

## DISCUSSION

In this study, we describe comprehensive genome-wide mapping of the interplay between TP53 and TP63 binding and the effects of different types of genotoxic stress in primary keratinocytes. These analyses reveal that TP53 and TP63 can bind to overlapping but distinct networks of binding sites, the specificity of which is determined through binding to differing TP53-like binding motifs. Importantly, our results suggest that constitutive TP53 binding sites are more strongly bound and contain more canonical-like TP53 response elements (TP53-RE), whereas those induced by DNA damage are weaker and more diverse and less well conserved. This suggests that induced TP53 binding is a distinct event, which correlates with recent evidence indicating that co-operative interaction between TP53 monomers is required for binding to low affinity sites associated with pro-apoptotic genes (60) and that this is important for the tumour suppressive capacity of TP53 (61). Furthermore, this also correlates well with recent data suggesting a two-step model of TP53 activation mediated through different combinations of half-sites (62).

The majority of TP53 sites we identified are bound by TP63 in untreated cells, irrespective of whether TP53 is constitutively bound or TP53 binding is induced by genotoxic stress. However, the converse is not true for TP63 sites and a large proportion of sites are not bound by TP53 in any of the conditions measured. Surprisingly, comparison of the changes in TP53 and TP63 binding upon cisplatin and adriamycin reveals substantial differences in the dynamics of both TP53 and TP63 binding, suggesting different mechanisms of regulation. Specifically, cisplatin treatment results in a substantial increase in both the number of sites and the amount of TP53 bound compared with adriamycin treatment.

This is in contrast to the effects on TP63 binding, which is greatly reduced on adriamycin treatment, compared with cisplatin treatment. Importantly, we also observed a significant number of strongly bound constitutive TP53 and TP63 sites in untreated cells, whereas TP53 sites induced *de novo* by genotoxic stress are generally weaker. Interestingly, these constitutive TP53 binding sites include canonical TP53 target genes CDKN1A, and RRM2B. Additionally, these canonical TP53/TP63 genes are up-regulated in a TP53-dependent manner in TP63 depleted cells, implying that removal of TP63-mediated repression is sufficient to activate TP53-mediated transcription. Irrespective of their constitutive or DNA-damage-induced nature, the majority of TP53 sites associated with DNA-damage-induced genes are also bound by TP63 in untreated cells and these are frequently associated with promoter proximal elements. Our genome-wide analyses identify >500 such ‘canonical’ targets and the results suggest and that >30% of TP53-dependent DNA damage-activated genes are associated with at least one constitutive TP53 binding site. This suggests that TP53 is pre-bound where it is poised for rapid activation in response to a stress signal, which is held in check through repression mediated by TP63 in unstressed cells.

Interestingly, ATM has been shown to both activate TP53 through serine-15 phosphorylation (63) and concomitantly phosphorylate ΔNP63α inactivating and targeting it for degradation (64). This may explain why we see a preference for TP53-induced targets activated in response to adriamycin treatment, compared with cisplatin treatment, since adriamycin is known to be a potent activator of ATM-mediated TP53 phosphorylation and concomitant stabilization (65), which may be sufficient to induce activation of a subset of constitutively bound genes. Importantly, TP53 is not stabilized to a significant extent in TP63 depleted cells, rather it is phosphorylated on serine-46 a post-translational modification associated with its transcriptional activation and also observed in response to adriamycin and cisplatin in this study. This highlights the importance of extending these studies to determine the co-factors and mechanisms involved in determining the transcriptional outcomes of the TP53/TP63 axis.

Our expression analyses revealed many more genes repressed in a TP53-dependent manner in response to genotoxic stress, in particular, to cisplatin treatment. These effects are likely influenced both directly through TP53-induced binding and indirectly through downstream effects of known TP53 targets CDKN1A or E2F7 (66) or indeed TP63 inactivation as demonstrated here. We did observe TP53 binding sites frequently associated with more distal TP53 binding events in enhancer-like elements the majority of which are associated with TP63 binding in untreated cells. Of course, as has now been shown for many transcription factors, ascribing functional significance of non-promoter associated binding events is more difficult. However, in contrast to induced genes, we identify a highly significant number of genes associated with only TP63 sites within 25 kb that are repressed in a TP53-dependent manner, suggesting that TP53 may elicit additional effects through modulating TP63 transcriptional activity. This is of particular interest, since we found that TP63 mRNA expression and protein levels are repressed in a TP53-dependent manner, concomitant with induced TP53 binding to the *TP63* C40 enhancer region (67). This additional level of control of TP63 activity by TP53 is particularly desirable in HFKs and other stratifying epithelia, as these tissues are exposed to high levels of environmental stress, such as UV radiation. Intriguingly, the 2236 genes we identify as down regulated in a TP53-dependent manner are enriched for cell cycle progression, DNA repair and metabolism, suggesting that in response to high levels of genotoxic stress TP53 can directly influence these processes through direct TP53 binding or indirectly through affecting TP63 activity.

Specifically, our downstream validation reveals novel roles for TP53 and TP63 in controlling a number of TP53-dependent DNA damage repressed repair genes. Expression of these genes is either directly repressed through TP53 binding (MSH2, XRCC4 and RAD51B) or repressed through a TP53-mediated alteration of TP63 activity (BRCA1, BRCA2 and FANCD2). This along with our genome-wide data indicates that in response to high levels of genotoxic stress TP53 activation results in a transcriptional shut down of a large number of DNA repair proteins in coordination with repression of cell cycle progression related genes. These binding events are opposed by TP63 and interestingly, TP53 repressive events are more frequently associated with more distal enhancer-like regions, the majority of which are bound by TP63 suggesting that these repressive effects may be influenced through modulating enhancer activity. This may be similar to recently described repressive effects elicited by TP53 binding to enhancer regions to repress, pluripotency genes in mouse embryonic stem cells (68).

Intriguingly, we noted that repressed DNA repair genes associated with only TP63, were enriched for critical regulators of homologous recombination and we show TP63 plays a role in the constitutive expression of BRCA1, BRCA2 and FANCD2 independent of TP53. Consequently, TP63 deficient HFKs are significantly impaired in their ability to repair double strand breaks induced by ionizing radiation irrespective of TP53-dependent effects on proliferation. In contrast, analyses of the genes identified as TP53-induced are greatly enriched for apoptosis, inhibition of cell cycle and epidermal/keratinocyte differentiation. Validation of these global observations with respect to DNA repair genes indicates different modes of TP53-mediated activation of a number of known TP53 activated repair genes (*RRM2B*, *DDB2* and *XPC*) (58,59,69), which can be repressed by TP63.

Interestingly, expression of DNA repair genes BRCA2, Rad51 and Mre11 have recently been shown to be activated by TP63 and TP73 in mouse embryonic fibroblasts (MEFS) (70) and it will therefore be important to extend these studies to other cells types which express a different complement of TP53 family members. Comparison of our data with recently published TP53 ChIP-seq data indicates significant overlaps, however, this is hard to interpret owing to the disparate cell types and damaging agents used (Supplementary Figure S15) (28,29,56,71), highlighting the need for further comprehensive systematic studies to further dissect the underlying mechanisms.

Together, our analyses reveal that through differential binding events, TP53 and TP63 coordinate expression of an extensive network of genes in response to genotoxic stress resulting in both transcriptional activation and repression. Loss of these repressive functions of TP53 or its counteraction through elevated TP63 expression has the potential to contribute to tumour growth and survival, in addition to loss of TP53's tumour suppressive pro-apoptotic and anti-proliferative functions. In support of this, when we compared our TP53/TP63 regulated genes with microarray data from tumours and matched normal in two independent head and neck squamous cell carcinoma cohorts (51,52), we found that there was significant enrichment for increased expression of genes that are TP53 repressed, whereas no significant enrichment was observed for TP53-induced genes. Importantly, most head and neck cancers contain mutant TP53 and increased levels of TP63, suggesting that maintenance of expression of these repressed genes is important in established tumours. In support of this, in a TP53 deficient mouse model of SCC, TP63 has recently been shown to be required for tumour survival through expression of FGFR2 (72), which we recently characterized as a TP63 target gene (26). Furthermore, TP63 repressive role likely extends beyond its functional opposition of TP53 target gene expression as evidenced by a recent study which showed that TP63 can repress target genes through SRCAP-mediated H2AZ deposition (73).

Using genome-wide approaches, these studies have revealed the scale of the interplay between TP53 and TP63 in normal HFKs and identified novel mechanisms of regulation, through which TP53 and TP63 coordinately influence expression of a vast array of genes. However, like most studies, this one is limited by the fact that we have not considered the function of TP73. While TP73 is expressed in very low levels in HFKs, they may be induced under certain conditions and impact functions of TP53 and TP63. However, our results do provide a framework to extend these analyses to other cellular and physiological settings considering the context of TP53 family members mutational status, isoform expression and post-translational modifications **t**o better determine how this network is de-regulated in cancer (reviewed in (74,75)). This is particularly pertinent, since recent observations have demonstrated that the canonical transactivation domain of TP53 have been shown to be, in part, dispensable for its tumour suppressive activity (23) and that mutant missense forms of TP53 have been shown to exert ‘gains of function’ through influencing TP63 and TP73 activity (17–19,76). As a result, a better understanding of how the TP53 family functions as a whole is critical for targeting this pathway in cancer.

## SUPPLEMENTARY DATA

Supplementary Data are available at NAR Online.

SUPPLEMENTARY DATA

## ACCESSION NUMBER

NCBI Gene Expression Omnibus accession number: GSE56640.
