# Supplementary material for: Genome-wide characterization reveals complex interplay between TP53 and TP63 in response to genotoxic stress
Source: Nucleic Acids Res. 2014 May 13;42(10):6270–85. doi: 10.1093/nar/gku299 (PMC4041465; doi:10.1093/nar/gku299)
Supplement: SUPPLEMENTARY DATA [file supp_42_10_6270__index.html]

Genome-wide characterization reveals complex interplay between TP53 and TP63 in response to genotoxic stress — SUPPLEMENTARY DATA 

# Genome-wide characterization reveals complex interplay between TP53 and TP63 in response to genotoxic stress

## SUPPLEMENTARY DATA

**Files in this Data Supplement:**

- SUPPLEMENTARY DATA
