# Supplementary material for: Genome-wide characterization reveals complex interplay between TP53 and TP63 in response to genotoxic stress
Source: Nucleic Acids Res. 2014 May 13;42(10):6270–85. doi: 10.1093/nar/gku299 (PMC4041465; doi:10.1093/nar/gku299)
Supplement: SUPPLEMENTARY DATA [file supp_gku299_nar-03118-x-2013-File008.zip › NAR-03118-2013 Suppl files/MCDADE_NAR__v6_Supplemental_with_legends_file_1-3.pdf]

Genome wide characterization reveals complex interplay between TP53 and TP63 in response to genotoxic stress.

Simon S McDade[1*], Daksha Patel[1], Michael Moran[1], James Campbell[2], Nicholas J Orr[2], Kerry Fenwick[2], Iwanka Kozarewa[2], Christopher J Lord[2], Alan A Ashworth[2], Dennis J. McCance[1*]

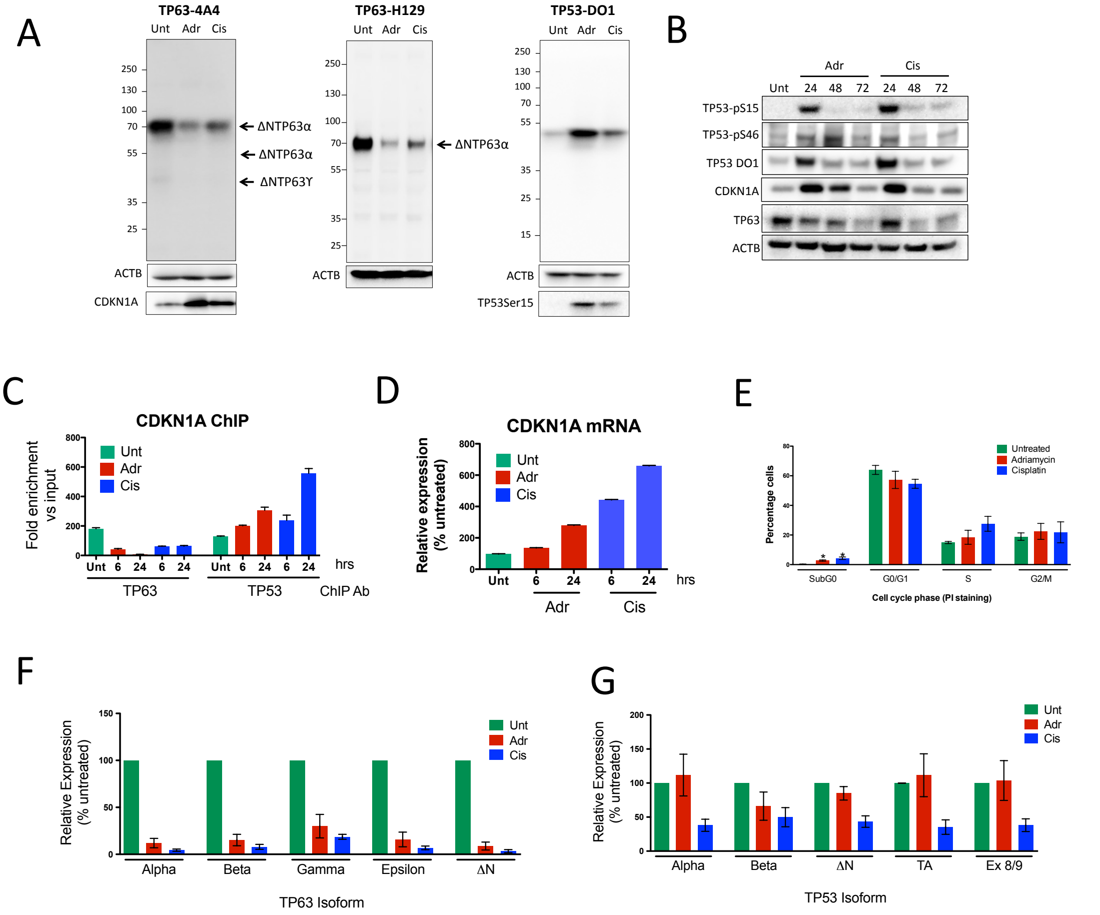

Figure S1: Characterisation of effects of adriamycin or cisplatin treatment on TP53/TP63 mediated regulation of the canonical target gene CDKN1A. (A) Whole membrane of western blot analysis for TP53 and TP63 antibody specificity in HFKs treated in the presence and absence of 350 nm adriamycin (Adr) or 25 µM Cisplatin (Cis) for 24 hours. (B) Western blot analysis of time course of adriamycin treatment at 24, 48 and 72hrs.
(C) Example of quantitative PCR of ChIP DNA prior to sequencing library prep on canonical TP53/TP63 bound CDKN1A promoter region comparing 6 and 24 hr treatment with untreated cells. (D) Quantitative PCR analysis of CDKN1A mRNA expression in response to genotoxic agent.
(E) FACS cell cycle analysis after 24hrs adriamycin and cisplatin treatment.
(F) Quantitative RT-PCR analysis of relative TP63 isoform expression. (G) Quantitative RT-PCR analysis of relative TP53 isoform.

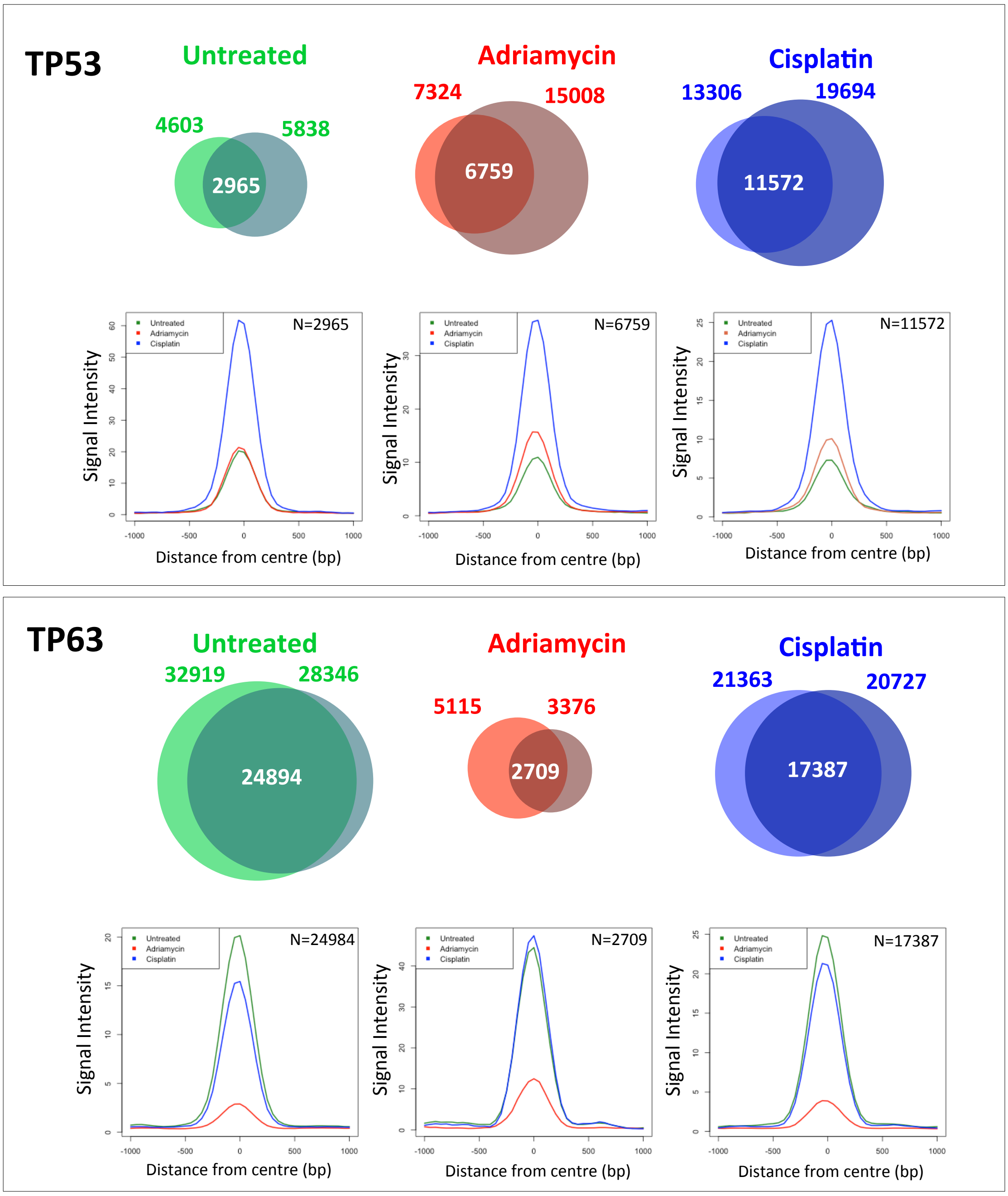

Figure S2: Generation of consensus peaksets. Venn diagrams illustrating overlap between ChIP-seq replicates for TP53 (top panel) or TP63 (bottom panel). These overlaps represent consensus peakset and are accompanied by plots of normalised binding intensity across each of consensus peakset for TP53 or TP63 respectively across the three treatment conditions.

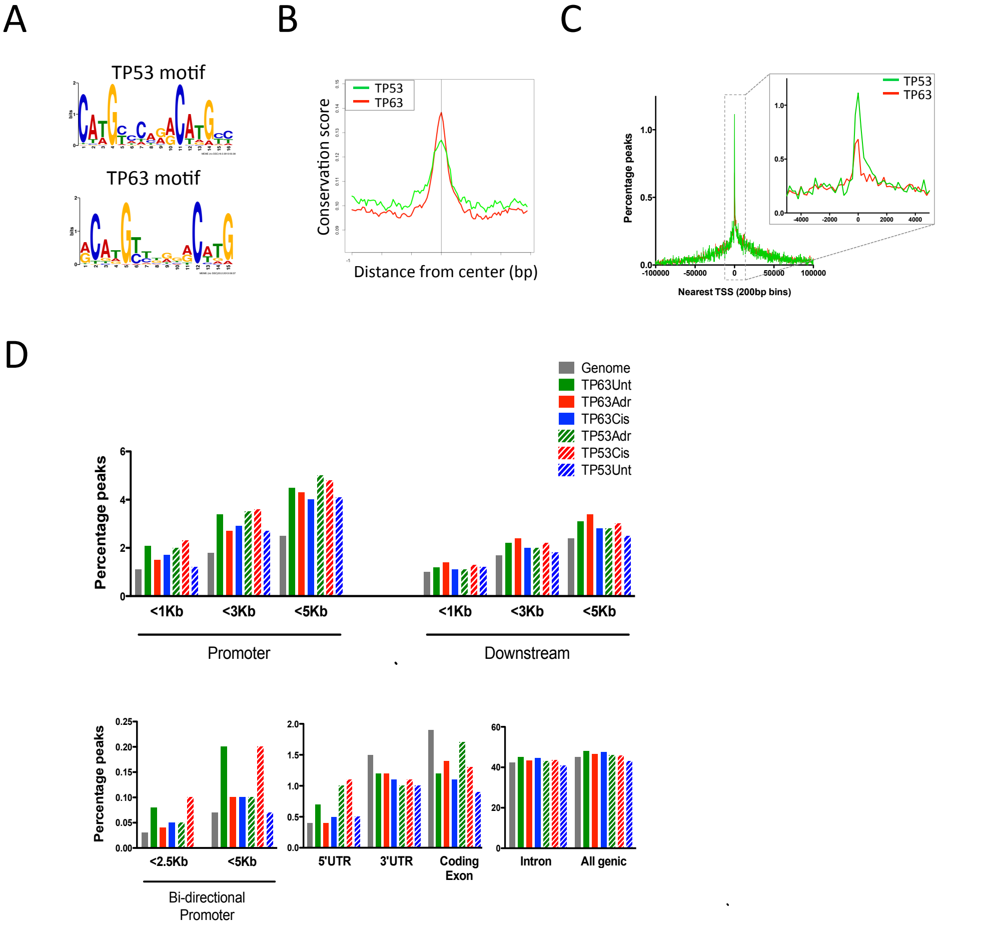

E

		Genome	TP63 Untreated peaks		TP63 Adriamycin peaks		TP63 Cisplatin peaks		TP53 Untreated peaks		TP53 Adriamycin peaks		TP53 Cisplatin peaks	
		%	% TP63 U peaks	p	%	p	%	p	%	p	%	p	%	p
Promoter	<1Kb	1.1	2.1	5.10E-47	1.5	0.007	1.7	7.70E-14	1.2	0.155	2	1.20E-01	2.3	3.80E-30
	<2Kb	1.8	3.4	2.00E-58	2.7	6.30E-04	2.9	1.10E-20	2.7	7.60E-04	3.5	8.50E-20	3.6	2.00E-37
	<3Kb	2.5	4.50E+00	4.50E-72	4.3	3.60E-04	4.00E+00	2.30E-32	4.10E+00	1.10E-07	5.00E+00	1.30E+31	4.80E+00	2.90E-48
Biprom	<2.5Kb	0.03	0.08	5.70E-05	0.04	0.229	0.05	1.50E-02	0	0.373	0.05	8.50E-02	0.1	4.40E-05
	<5Kb	0.07	0.2	1.20E-07	0.1	0.047	0.1	4.80E-04	0.07	0.545	0.1	1.30E-01	0.2	3.40E-05
Downstream	<1Kb	1	1.2	3.10E-04	1.4	0.021	1.1	2.40E-02	1.2	0.112	1.1	2.09E-01	1.3	8.60E-04
	<2Kb	1.7	2.2	3.70E-07	2.4	0.004	2	0.0.005	1.8	0.35	2	4.50E-02	2.2	5.50E-05
	<3Kb	2.4	3.1	1.00E-11	3.4	5.50E-04	2.8	3.90E-04	2.5	3.20E-01	2.8	2.30E-02	3	6.90E-05
Gene	5' UTR	0.4	0.7	2.00E-08	0.4	0.417	0.5	2.30E-04	0.5	0.325	1	1.40E-09	1.1	2.70E-19
	3' UTR	1.5	1.2	8.70E-05	1.2	0.179	1.1	1.10E-05	1	0.025	1	1.00E-03	1.1	4.50E-05
	Coding Exon	1.9	1.2	2.70E-20	1.4	0.013	1.1	1.90E-20	0.9	5.00E-05	1.7	7.50E-02	1.3	1.50E-07
	Intron	42.4	45	1.30E-17	43.5	0.122	44.8	2.20E-11	40.7	0.032	43.1	9.50E-02	43.4	1.40E-02
	All	45.2	48.1	1.50E-09	46.5	0.379	47.5	1.00E-04	43.1	3.00E-04	45.9	1.43E-01	45.8	9.70E-02

Figures S3: Characterisation of pooled TP53 and TP63 binding sites. (A) *De-novo* motifs identified from 500bp sequences centered at the midpoint of 5000 randomly sampled TP53 or TP63 binding sites reveals similar but distinct motifs. (B) Conservation plots of vertebrate Phastcons scores for region +/- 3Kb around midpoint of all 12,287 TP53 or TP63 binding sites. (C) Plot of distance to nearest TSS from midpoint of TP53 or TP63 binding sites. (D and E) Analysis of enrichment peak location of consensus peaksets with reference to genomic features generate using the Cis-regulatory Element Annotation System (CEAS) analysis tool.

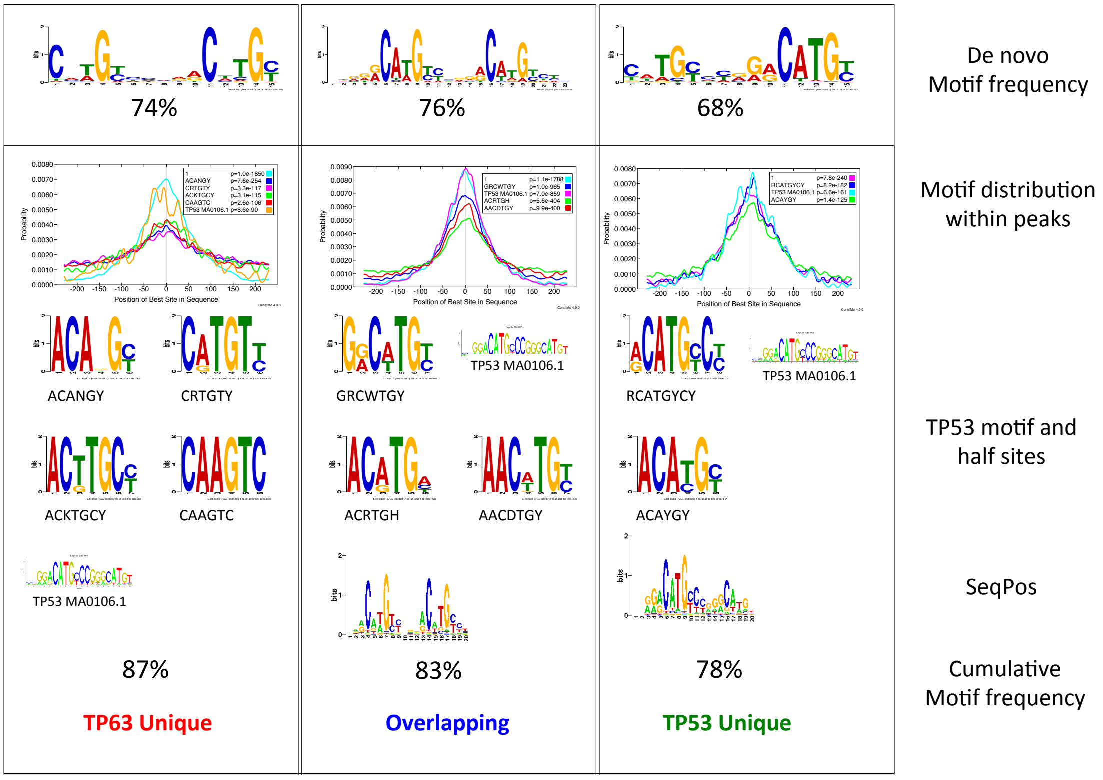

Figure S4: TP53 and TP63 exhibit differential motif binding preferences. Combined *de-novo* (MEME-ChIP) (Machanick and Bailey 2011) and motif enrichment analysis of TP63 unique, overlapping and TP53 unique subsets of sites identified in Fig. 3F reveals that at least one TP53/TP63 site or half is present in the majority of binding sites. Analysis was carried out on the 500 Bp surrounding the peak midpoint for all 2042 TP53 unique peaks and 5000 randomly selected TP63 unique and overlapping peaks.

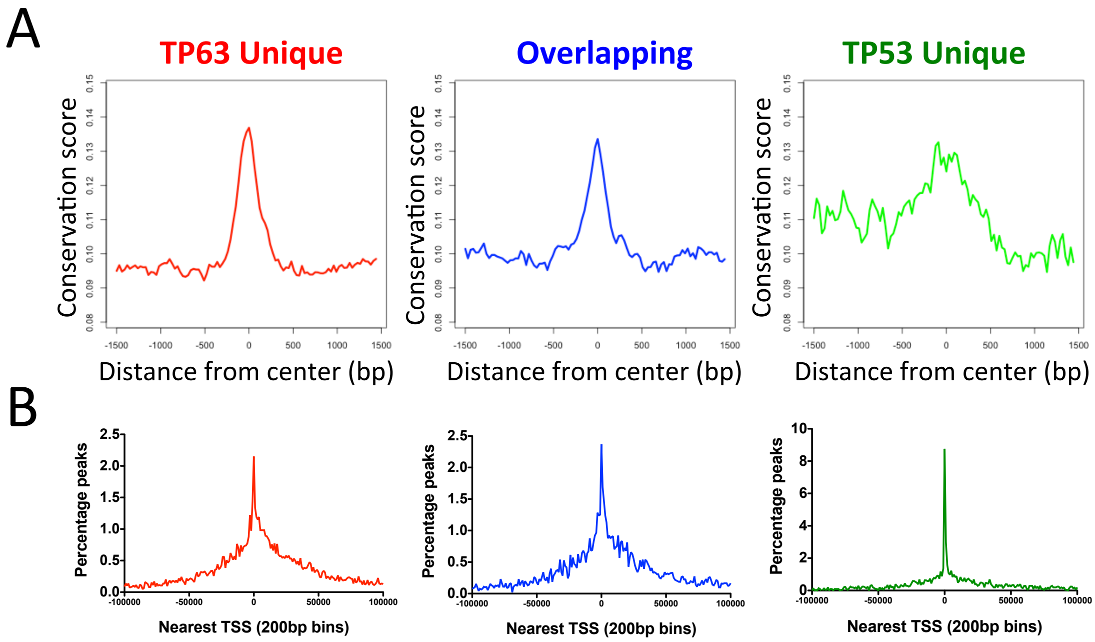

Figure S5: Characterisation of TP63 unique, Overlapping and TP53 unique peaksets. (A) Conservation plots of vertebrate Phastcons scores for region +/- 3Kb around peaks centre. (B) Plot of distance to nearest Refseq transcription start site (TSS).

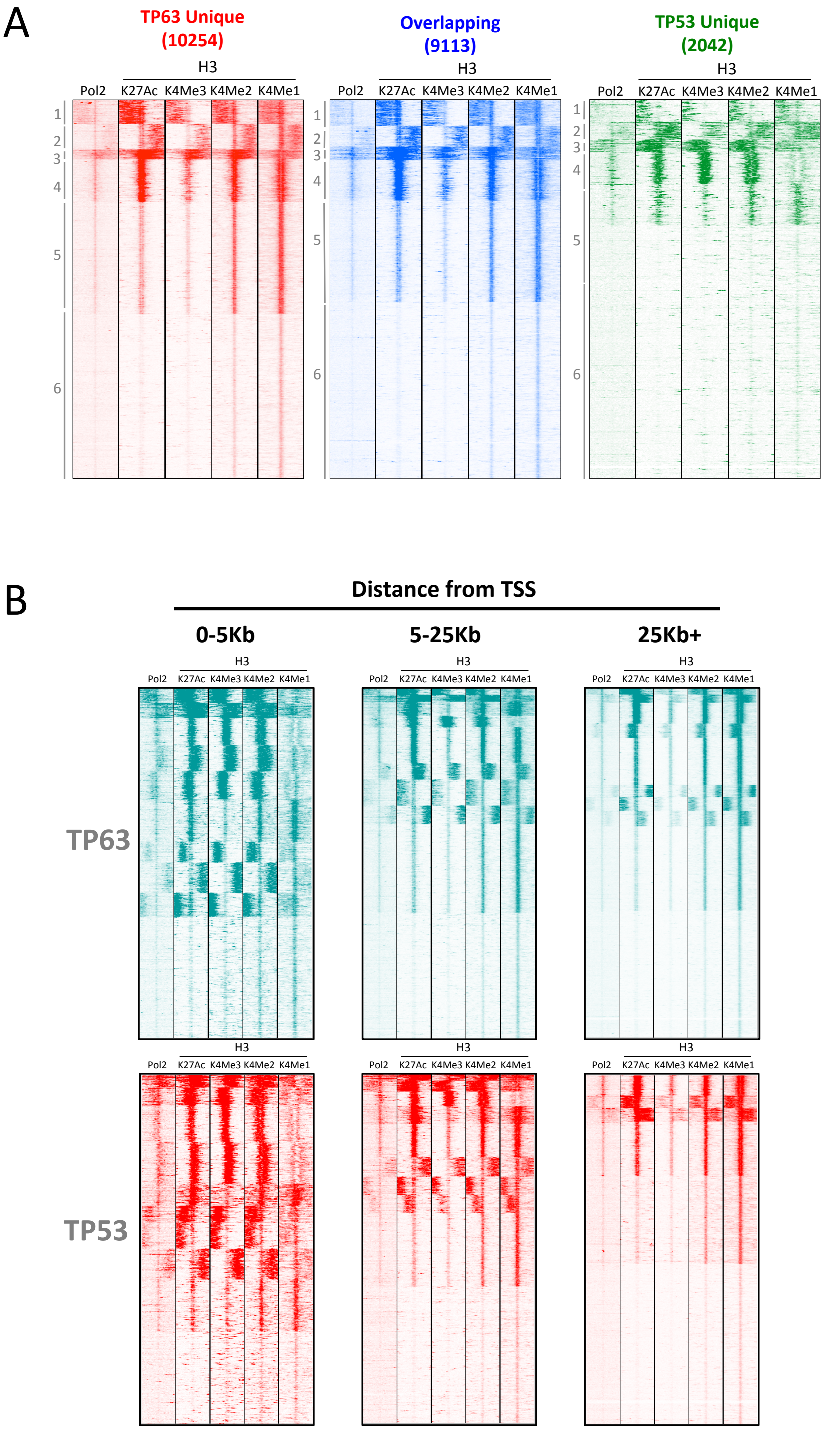

Figure S6: Characterisation of histone modifications in regions surrounding TP63 unique, overlapping and TP53 unique peaksets. (A) Mean density plots of the 10Kb region surrounding the overlapping and TP53 and TP63 unique peaksets (Fig. 3A) of RNApol-II, H3K27 Acetylation, H3K4 mono -, di- and tri-methylation. Histone modification data from Broad Encode histone modification data in Normal Human Epidermal Keratinocytes (NHEK) (ENCODE Project Consortium 2011). All plots and K-Means clustering generated using Seqminer (Ye et al. 2011). (B) Similar plots for TP63 and TP53 sites classified by proximity to nearest Refseq transcription start site (TSS).

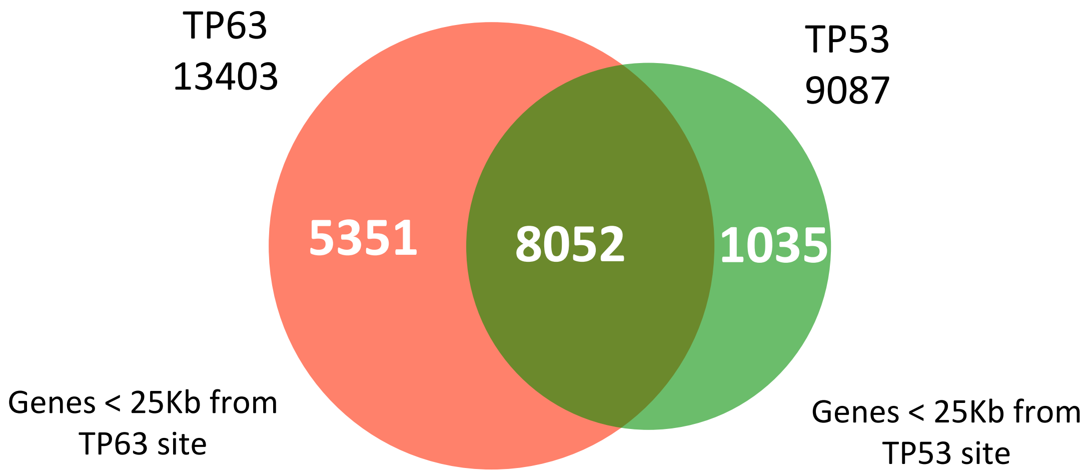

Figure S7: Annotation of peaks to Refseq genes. Venn diagram comparing binding sites annotated to Refseq genes within 25Kb of a TP53 or TP63 binding sites.

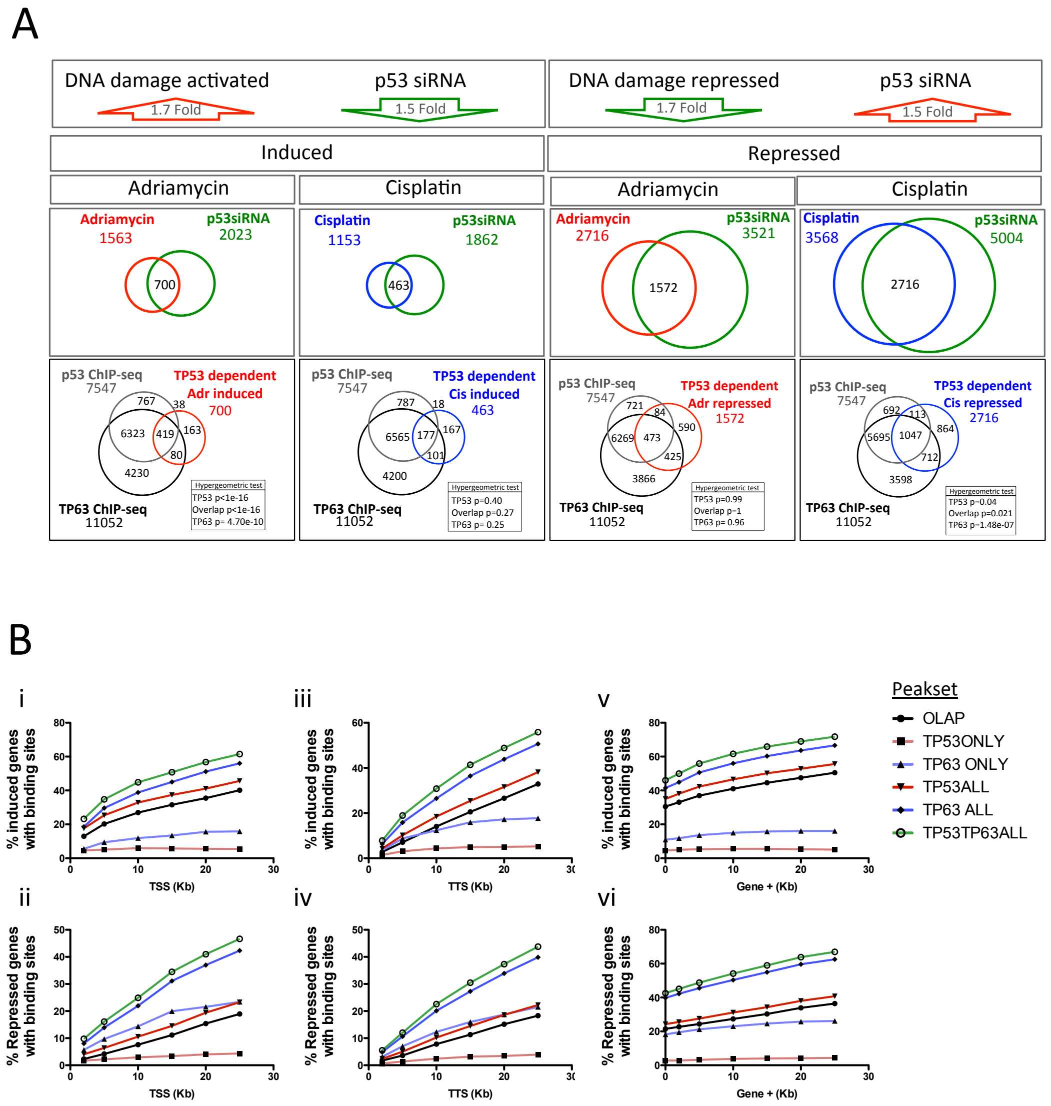

Figure S8: Microarray data analysis. (A) Genes were deemed to be induced or repressed if they were up/down-regulated 1.7-fold in response to either adriamycin or cisplatin. This was deemed to be TP53 dependent if this change was reversed 1.5-fold in cells depleted for TP53. To identify potential directly regulated target genes, these TP53 dependently regulated genes were then compared with those within 25Kb of a TP53 or TP63 binding site. (B) Relationship between induced and repressed genes and presence of TP63 or TP53 binding site within specified distance of Refseq transcription start site (TSS)(i and ii), transcription termination site (TTS)(iii and iv) or the genbody (v and vi)

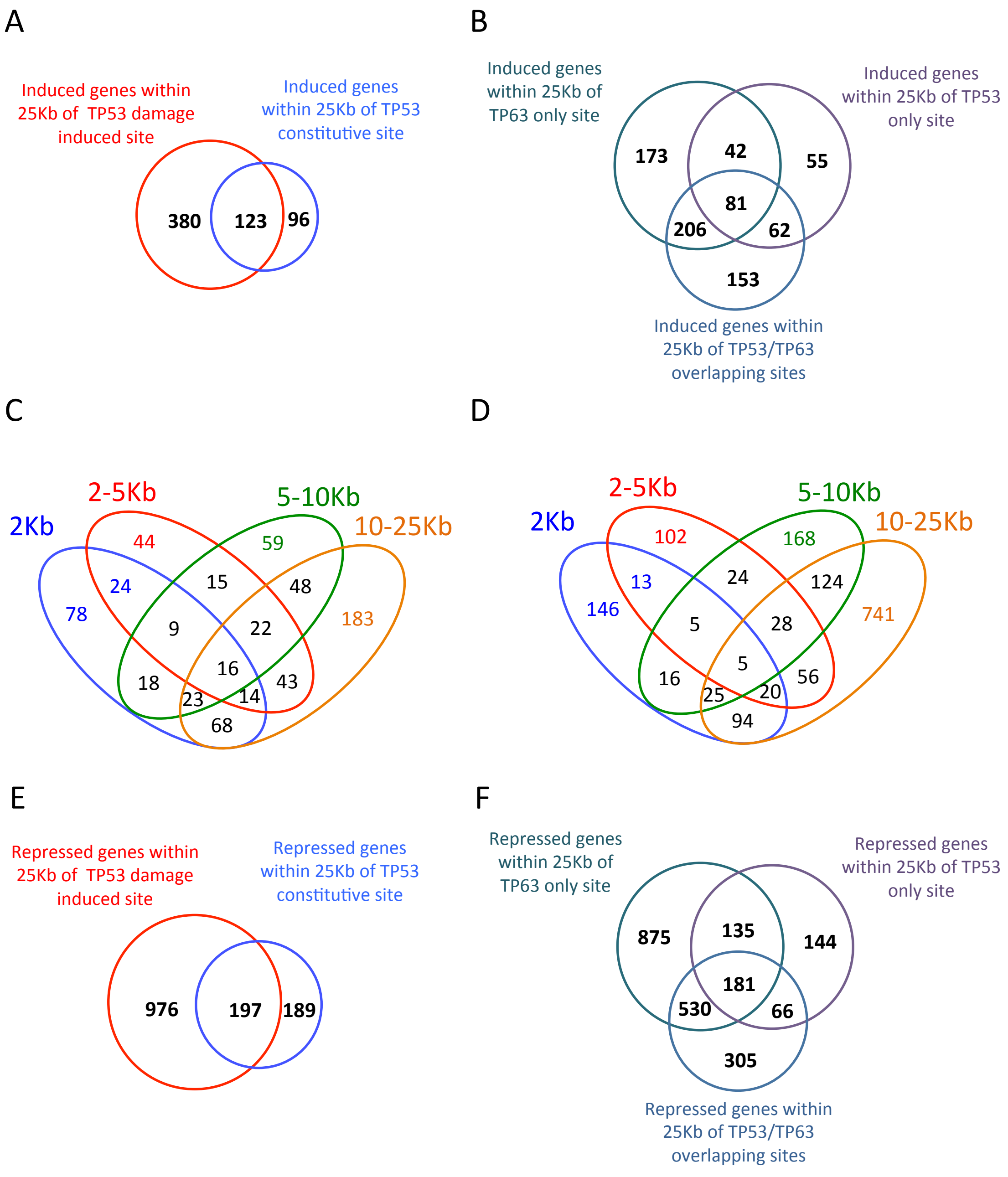

Figure S9: (A) Comparison of binding sites associated with TP53 bound damage induced genes and those already TP53 bound in untreated cells (n=599 genes). (B) Comparison of number of damage induced genes associated with TP53 only, TP63 only and TP53/TP63 overlapping binding sites (n=772 genes). Characterisation of location relative to nearest transcription start site (TSS) of TP53/TP63 binding sites relative to induced (C) or repressed (D) genes. (E) Comparison of Binding sites associated with TP53 bound damage repressed genes and those already TP53 bound in untreated cells (n=1362 genes). (F) Comparison of number of repressed genes associated with TP53 only, TP63 only and TP53/TP63 overlapping binding sites (n=2236).

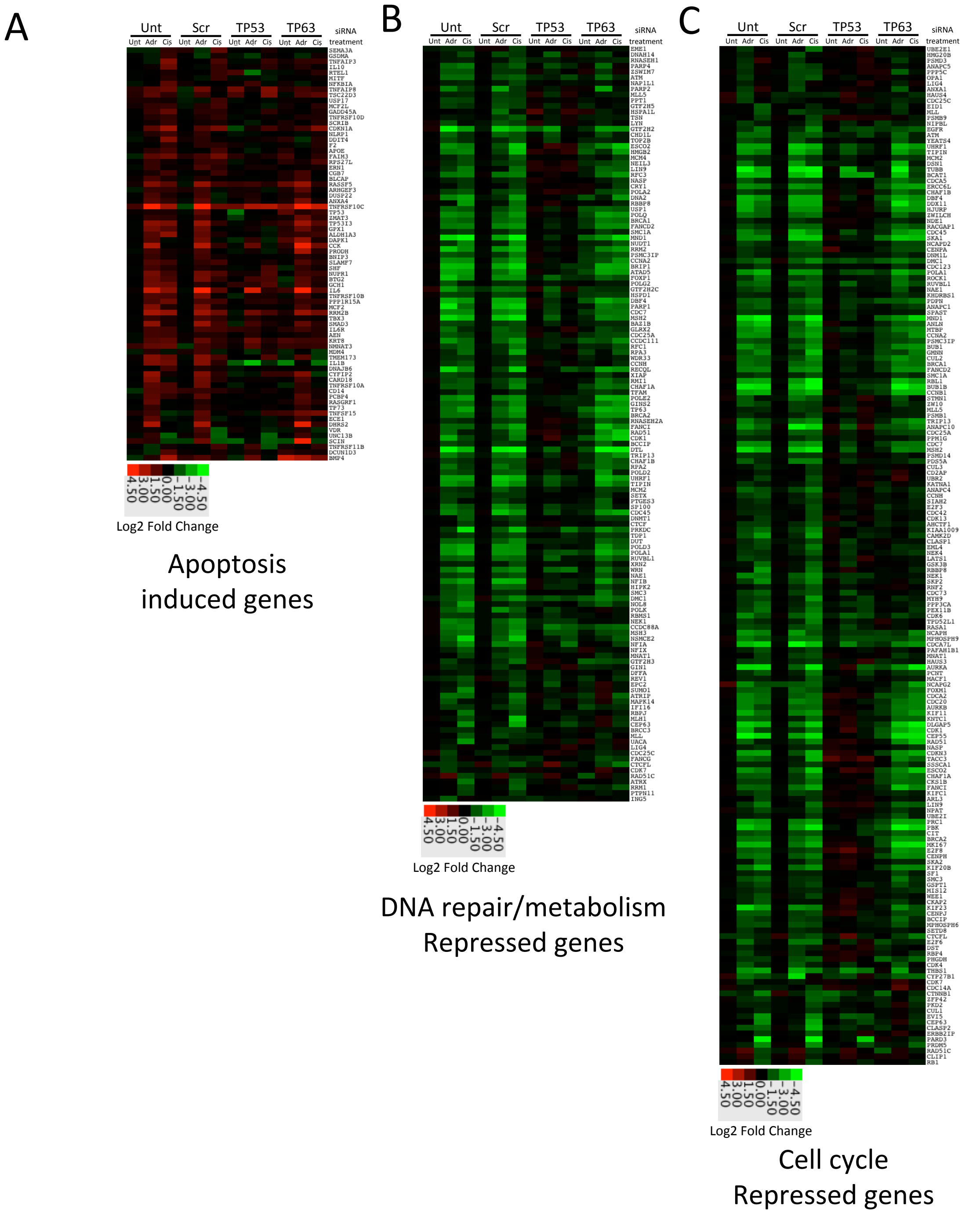

Figure S10: Clustering of microarray expression values for examples of gene ontology analysis. (A) Induced genes associated with apoptosis. (B) Repressed genes associated with DNA repair and metabolism. (C) Repressed genes associated with cell cycle. Microarray data for genes associated with each GO was extracted and clustered with Cluster 3.0 software (de Hoon et al. 2004) and visualised using Java TreeView (Saldanha 2004).

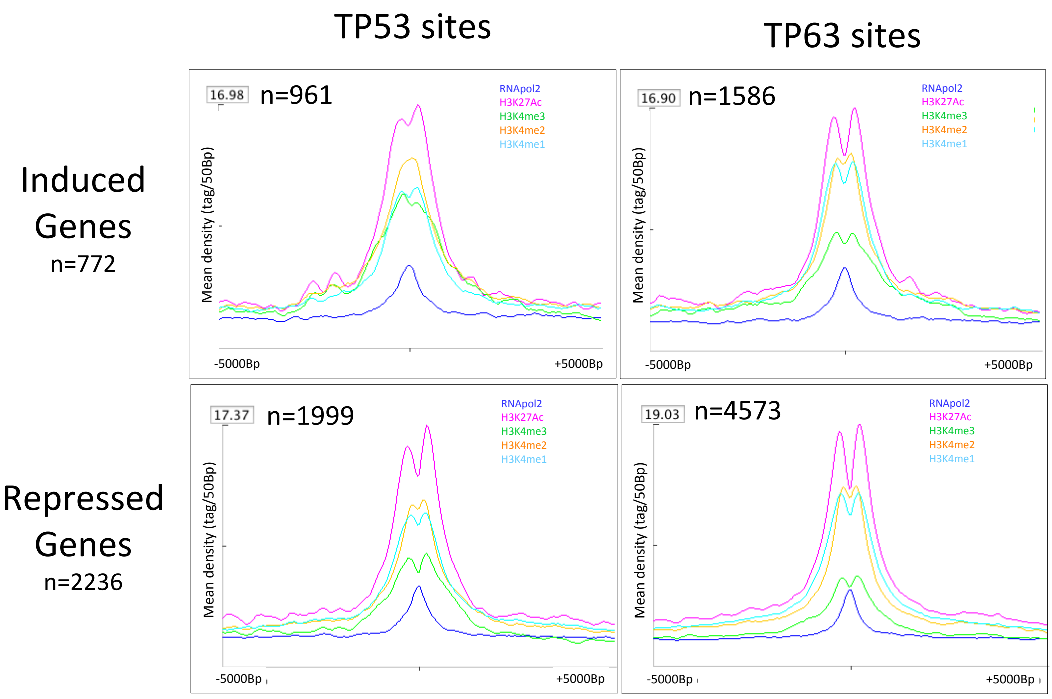

Figure S11: Histone modification profiles in normal human epidermal keratinocytes in regions surrounding TP53 and TP63 sites within 25Kb of target genes. Mean density plots of ChIP-seq histone modifications and RNApol-II signal for the 10Kb region surrounding the TP53 and TP63 binding sites within 25Kb of 777 TP53 dependently induced and 2236 repressed genes. Profiles generated using Seqminer (Ye et al. 2011) based analysis of Broad Encode histone modification data in Normal Human Epidermal Keratinocytes (NHEK).

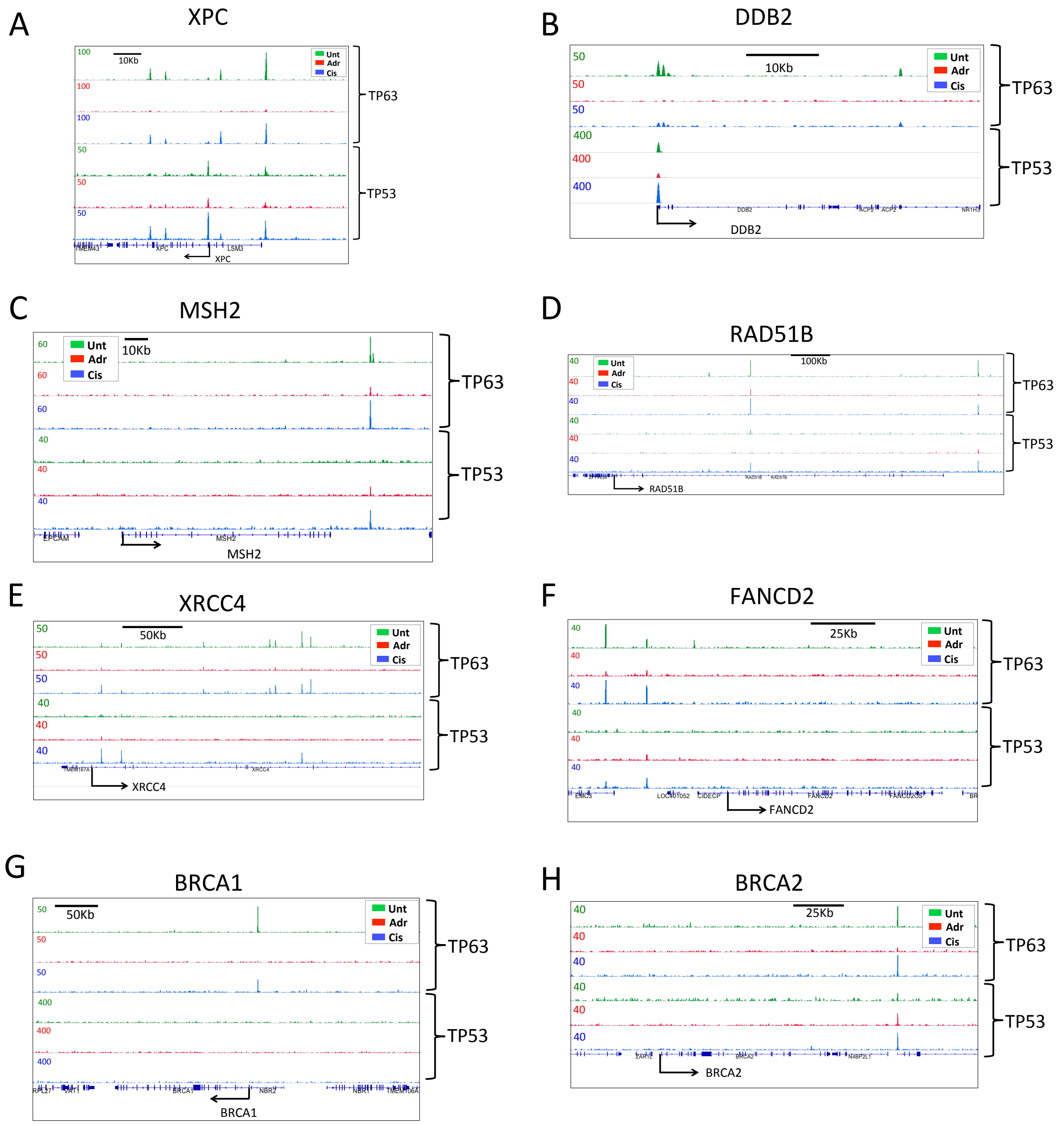

Figure S12: Visualisation of binding sites associated with TP53 dependently induced and repressed DNA repair genes.
Normalised ChIP-seq biological replicate binding profiles in the presence and absence of genotoxic agents adriamycin (350nM) and cisplatin (25 μM) for: 1) induced genes associated with TP53 and TP63 binding within 25Kb (A-B); 2) repressed genes associated with TP53 and TP63 binding within 25Kb (C-E) Repressed genes associated with TP63 binding only within 25Kb (F-H).

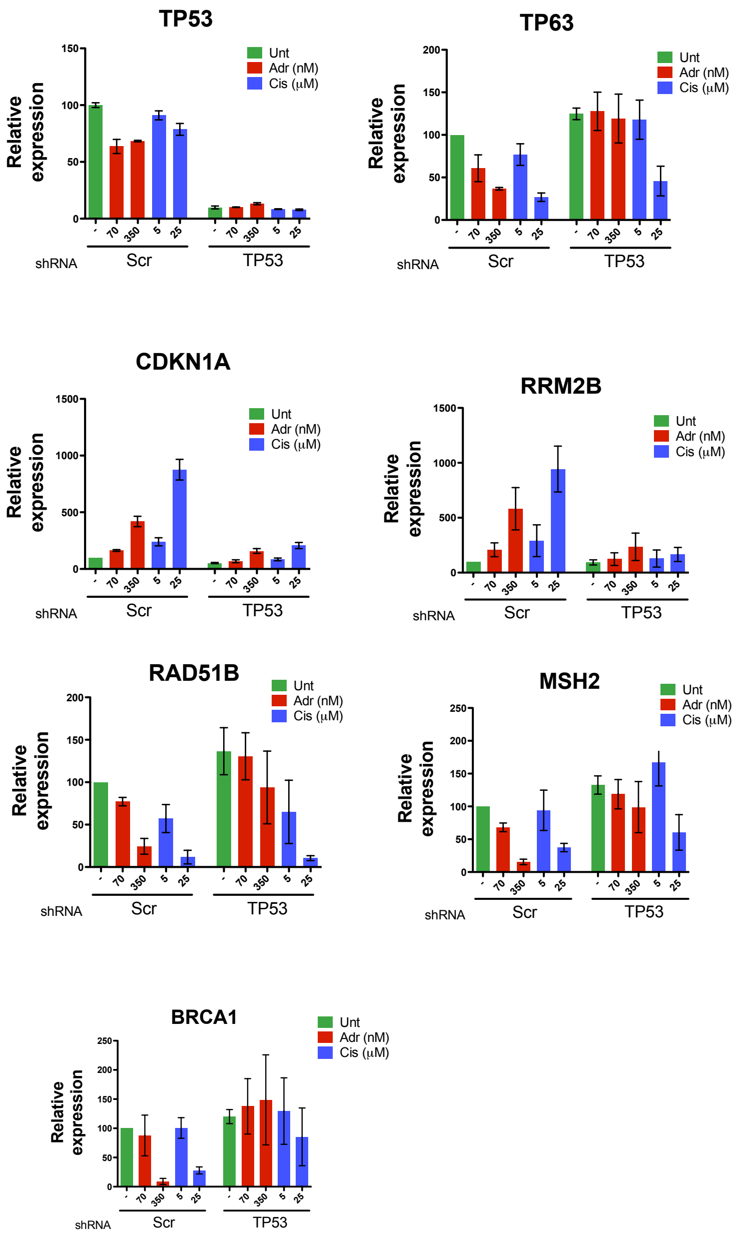

Figure S13: Additional validation of effects of transient and stable TP53 depletion on transcription of DNA repair target genes. qRT-PCR quantification of TP53, TP63 and various target genes mRNA in HFKs stably depleted for TP53 in response to treatment with increasing amounts of adriamycin (70 nM/350 nM) or cisplatin (5 μM/25 μM). Graphs show mean +/- SEM of three biological replicates.

Thurlow et al.	p53 induced genes (p53/p63 bound) 773	p53 repressed genes (p53/p63 bound) 2236
HNSCC Increased 1840	72<br>ns	**473**<br>p < 1e-16
HNSCC Decreased 1528	67<br>ns	234<br>(p=0.014)

Pyeon et al.	p53 induced genes (p53/p63 bound) 773	p53 repressed genes (p53/p63 bound) 2236
HNSCC Increased 1528	60<br>ns	**414**<br>p < 1e-16
HNSCC Decreased 956	48<br>ns	154<br>(p=0.0082)

Figure S14: **TP53 dependently repressed genes are elevated in head and neck squamous cell (HNSCC) carcinoma microarray datasets.** Analysis of two publically available HNSCC array datasets, comparing tumour with normal indicates that TP53 dependently repressed, TP53/TP63 bound genes are significantly elevated in HNSCC. Differentially expressed genes between tumour and normal (+/-1.5fold, p<0.05) were detected by three- way ANOVA taking into account site and HPV status.

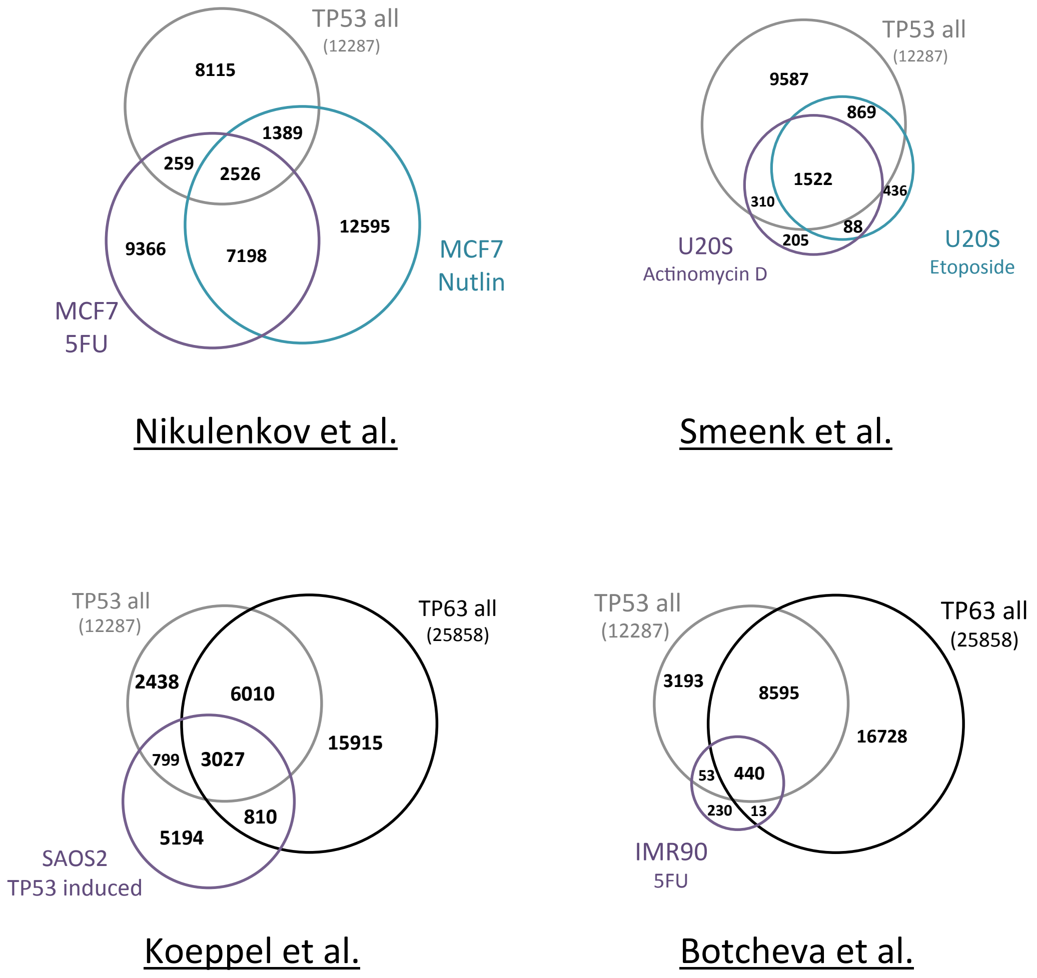

Figure S15: Comparison of ChIP-seq data with published TP53 ChIP-seq studies. Venn diagrams illustrating overlap of data from our study with TP53 ChIP-seq results. (A) MCF7 cells treated with 5FU or nutlin-3 (Nikulenkov et al. 2012) . (B) U2OS cells treated with actinomycin D or etoposide (Smeenk et al. 2011). (C) TP53 induced in SAOS2 cells (Koeppel et al. 2011). (D) IMR90 cells treated with 5FU (Botcheva et al. 2011).
